# Stable Chitosan-Based Nanoparticles Using Polyphosphoric Acid or Hexametaphosphate for Tandem Ionotropic/Covalent Crosslinking and Subsequent Investigation as Novel Vehicles for Drug Delivery

**DOI:** 10.3389/fbioe.2020.00004

**Published:** 2020-01-24

**Authors:** Ramzi Mukred Saeed, Isra Dmour, Mutasem O. Taha

**Affiliations:** ^1^Department of Pharmaceutical Sciences, Faculty of Pharmacy, University of Jordan, Amman, Jordan; ^2^Faculty of Pharmacy and Medical Sciences, Al-Ahliyya Amman University, Amman, Jordan; ^3^Department of Pharmaceutical Chemistry and Pharmacognosy, Faculty of Pharmacy, Applied Science Private University, Amman, Jordan

**Keywords:** chitosan, ionotropic gelation, polyphosphoric acid, hexametaphosphate, phosphoramide bond, doxorubicin

## Abstract

Chitosan nanoparticles (NPs) are widely studied as vehicles for drug, protein, and gene delivery. However, lack of sufficient stability, particularly under physiological conditions, render chitosan NPs of limited pharmaceutical utility. The aim of this study is to produce stable chitosan NPs suitable for drug delivery applications. Chitosan was first grafted to phthalic or phenylsuccinic acids. Subsequently, polyphosphoric acid (PPA), hexametaphosphate (HMP), or tripolyphosphate (TPP) were used to achieve tandem ionotropic/covalently crosslinked chitosan NPs in the presence of 1-ethyl-3-(3-dimethylaminopropyl)-carbodiimide (EDC). Thermal and infrared traits confirmed phosphoramide bonds formation tying chitosan with the polyphosphate crosslinkers within NPs matrices. DLS and TEM size analysis indicated spherical NPs with size range of 120 to 350 nm. The generated NPs exhibited excellent stabilities under harsh pH, CaCl_2_, and 10% FBS conditions. Interestingly, DLS, NPs stability and infrared data suggest HMP to reside within NPs cores, while TPP and PPA to act mainly as NPs surface crosslinkers. Drug loading and release studies using methylene blue (MB) and doxorubicin (DOX) drug models showed covalent PPA- and HMP-based NPs to have superior loading capacities compared to NPs based on unmodified chitosan, generated by ionotropic crosslinking only or covalently crosslinked by TPP. Doxorubicin-loaded NPs were of superior cytotoxic properties against MCF-7 cells compared to free doxorubicin. Specifically, DOX-loaded chitosan-phthalate polyphosphoric acid-crosslinked NPs exhibited 10-folds cytotoxicity enhancement compared to free DOX. The use of PPA and HMP to produce covalently-stabilized chitosan NPs is completely novel.

## Introduction

Chitosan (C) is a semisynthetic polyaminosaccharide obtained by N-deacetylation of chitin. Chitosan has attracted attention in various biomedical, pharmaceutical, food, and environmental fields due to its safe profile, biodegradability, and biocompatibility, in addition to its bacteriostatic and mucoadhesive properties (Alves and Mano, 2008; Riva et al., 2011; Miola et al., 2015; Silva et al., 2017; Bracharz et al., 2018; Dmour and Taha, 2018; Jiang and Wu, 2019; Savin et al., 2019).

**Figure 1:**
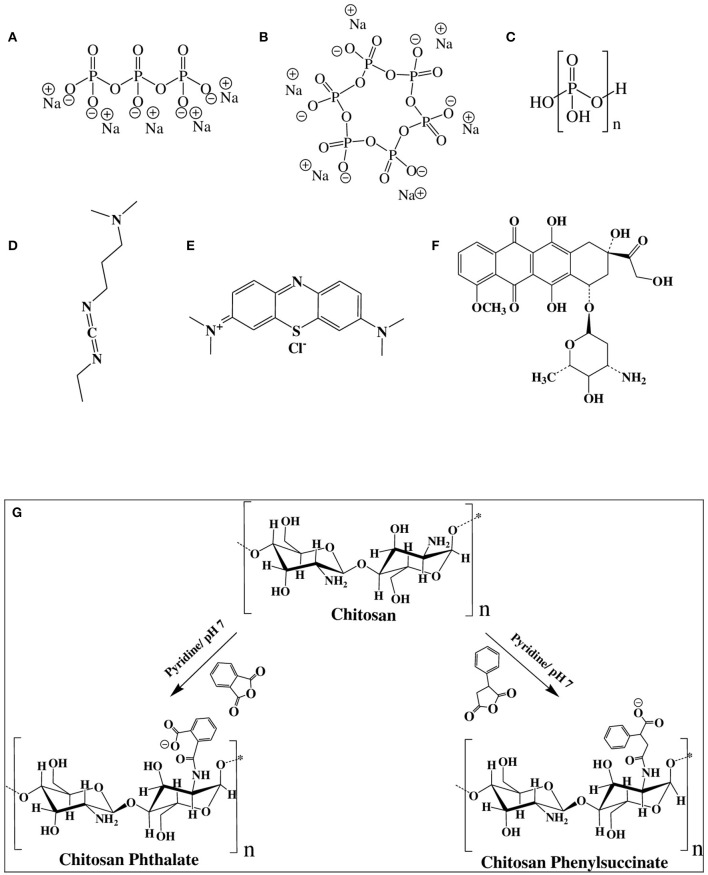
Chemical structures of **(A)** sodium tripolyphosphate (TPP), **(B)** sodium hexametaphosphate (HMP), **(C)** polyphosphoric acid (PPA), **(D)** 1-ethyl-3-(3-dimethylaminopropyl) carbodiimide (EDC), **(E)** methylene blue (MB), **(F)** doxorubicin (DOX), and **(G)** Chemistry of grafting chitosan to phthalic and phenylsuccinic acids.

Chitosan nanoparticles (NPs) are widely studied as nanocarriers for drug, protein, and gene delivery systems (Almaaytah et al., 2018; Baghdan et al., 2018). Ionotropic gelation is the most studied formulation method for preparing chitosan NPs. It is based on electrostatic interaction between the positively-charged aminosugar monomeric units of chitosan and negatively-charged polyanions, e.g., tripolyphosphate (TPP, Figure 1A) or hexametaphosphate (HMP, Figure 1B), or dextran sulfate (Katas et al., 2013; Kiilll et al., 2017; Rassu et al., 2019). Although ionotropic chitosan NPs have many benefits as drug delivery systems, there are still many barriers to be resolved to realize their clinical potential. These include inadequate oral bioavailability, instability in blood circulation, and toxicity (Du et al., 2014).

NPs sizes and surface charges have significant implications on their biological properties such as cellular uptake and biodistribution *in vivo*. Nanoparticles of diameters ranging from 10 to 300 nm have been reported to cross the gaps in blood vessels supplying tumor cells without significant penetration to healthy tissues (Grossman and McNeil, 2012; Yan et al., 2015). Similarly, NPs with slight negative charges, i.e., under physiological pH, tend to accumulate in tumor cells more efficiently (Honary and Zahir, 2013).

HMP is non-toxic substance, widely used in food industry as a sequestering agent and food additive (Baig et al., 2005; Parab et al., 2011). HMP was also used as stabilizer of BaSO_4_ (Gupta et al., 2010) and ZnCdS (Wang et al., 2011b) nanoparticles. Additionally, TPP and HMP have been reported as ionotropic crosslinking agents for the preparation of chitosan NPs for drug delivery purposes (Nair et al., 2019; Rassu et al., 2019; Su et al., 2019).

On the other hand, although polyphosphoric acid (PPA, Figure 1C) was never used as ionotropic crosslinking agent for the preparation of NPs, chitosan-PPA beads (microspheres) have been used as a delivery system for proteins and peptides (Yuan et al., 2018). PPA-coated NPs were also used for blood pool imaging *in vivo* (Peng et al., 2013).

Carbodiimide coupling agents, in particular, EDC (Figure 1D), have been utilized to immobilize enzymes on chitosan NPs to enhance enzymatic stability in solution (Sun et al., 2017). Additionally, EDC is useful for surface crosslinking/immobilization of medicinal compounds onto NPs to enhance their stabilities to variable pH or temperature conditions (Shen et al., 2009; Chaiyasan et al., 2015; Esfandiarpour-Boroujeni et al., 2016; Song et al., 2018). EDC was also used for covalent crosslinking and stabilization of doxorubicin-loaded chitosan-TPP NPs (Dmour and Taha, 2017), lutein-loaded chitosan-dextran NPs (Chaiyasan et al., 2015) and doxorubicin-loaded PEG-PLGA nanoparticles (Luo et al., 2019).

In this investigation, we describe the use of polyphosphoric acid (PPA) and sodium hexametaphosphate (HMP), for the first time, as tandem ionotropic/covalent crosslinkers for stabilizing chitosan-phthalate- and chitosan-phenylsuccinate based NPs in the presence of EDC.

HMP and PPA provide more anionic charges per molecule compared to TPP, as in Figure 1, which should offer more interaction sites for ionotropic crosslinking with chitosan's cationic ammonium groups. Moreover, HMP and PPA are non-toxic, and therefore, superior to covalent crosslinkers such as glutaraldehyde, genipin, and glyoxal, which tend to exhibit significant toxicities (Dmour and Taha, 2018). The resulting NPs were characterized vis-à-vis their size ranges, surface charges, and physical stabilities under harsh pH, CaCl_2_, and FBS conditions. The generated NPs exhibited excellent stabilities under such conditions. Drug loading and release studies using methylene blue (MB, Figure 1E) and doxorubicin (DOX, Figure 1F) model drugs showed covalent PPA- and HMP-based NPs to have superior loading capacities and release profiles. DOX-loaded NPs showed enhanced cytotoxic properties compared to free doxorubicin.

## Materials and Methods

### Materials

All chemicals were purchased from respective companies (in brackets) and were used without pretreatment or purification. Pyridine, absolute ethanol, and acetone of analytical grades (Carlo Erba France, and Labchem, USA). Medium molecular weight chitosan, phenylsuccinic anhydride, and sodium hexametaphosphate (HMP) (Sigma-Aldrich, USA). Polyphosphoric Acid (PPA) and phthalic anhydride (Fluka, Switzerland). Ultrapure water (conductivity = 0.05 μs/cm) for DLS size analysis (Millipore, USA).

Penta basic sodium tripolyphosphate (Sigma-Aldrich, Germany), N-ethyl-N′-(3-dimethylaminopropyl) carbodiimide hydrochlorides (EDC) (Sigma- Aldrich, USA), hydrochloric acid (37%) (Carlo Erba, Spain) and sodium hydroxide (Rasayan Laboratory, India). Dialysis tubing (molecular weight cutoff = 14 kDa, Sigma-Aldrich, USA), Tris base buffer (Bio Basic Inc., Canada), Methylene blue (Seelze, Germany), and Doxorubicin HCl (Ebwe Pharma, Austria). CellTiter Non-Radioactive Cell Proliferation Assay Kit from Promega (USA). RPMI 1640 medium and fetal bovine serum (FBS) were purchased from (Caissan, USA), L-glutamine, penicillin–streptomycin and trypsin-EDTA were purchased from (EURO Clone, Italy). 4′,6-Diamidino-2-phenylindole (DAPI) stain was purchased from Invitrogen (Thermo Fisher Scientific, USA). Poly-L-lysine obtained from (Sigma- Aldrich, Germany).

### Synthesis of Chitosan-Dicarboxylic Acid Derivatives and Preparation of Corresponding NPs

Chitosan-dicarboxylic acid derivatives (chitosan-phthalate and chitosan phenyl succinate) were prepared as described earlier (Aiedeh and Taha, 1999) with slight modifications. Briefly, chitosan (1.00 g, corresponding to 5.58 mmol glucosamine) was dissolved in (50 ml) HCl (0.37% v/v) aqueous solution at ambient temperature. The particular anhydride (phthalic or phenylsuccinic acids, 2.5 or 5.0 mmol, respectively) was dissolved in (5 ml) pyridine and added dropwise to chitosan solution with vigorous stirring. NaOH (1.0 M) solution was added dropwise to the reaction mixture to maintain reaction pH at 7.0. The reaction was allowed to continue for 40 min. Subsequently, the resulting chitosan derivative was precipitated by gradual addition of acetone under continuous stirring. The resulting precipitate was filtered, washed three times with absolute ethanol (100 ml), and finally with acetone (100 ml), and dried for 48 h in hot air oven at 35°C. The products were stored in airtight bottles.

Chitosan- and chitosan-carboxylate-based NPs were prepared using “syringe method” as described earlier (Calvo et al., 1997). Briefly, chitosan, or chitosan derivative, was dissolved by stirring for 48 h in aqueous HCl (4.8 mM) to produce 0.1%w/v solution. Wherever needed, the resulting solution was filtered or centrifuged at 4,000 rpm for 10 min at 25°C to remove any insoluble polymer residues. Subsequently, freshly prepared crosslinker aqueous solution, namely TPP (0.4%w/v), PPA (0.2%w/v activated by heating at 100°C for 1.5 h), or HMP (0.1% w/v) was added gradually, using syringe, to prepared chitosan, or chitosan-carboxylate solutions (5 ml) under vigorous magnetic stirring at 25°C until visual appearance of opalescent hazy dispersion (representing NPs formation). The resulting NPs were used for size and zeta potential analysis (i.e., dynamic light scattering, DLS) purposes without further processing.

The prepared NPs were covalently crosslinked using EDC as described earlier (Dmour and Taha, 2017). Briefly, EDC (25 mg) was added to NPs dispersions (5 ml) prepared from chitosan or its derivatives by ionotropic gelation method. The reaction mixture was stirred vigorously over 1 min, and then it was allowed to stand for 1 h. Subsequently, the reaction was terminated by one of the following ways: (1) For size, zeta potential and stability analysis purposes (which require minute amounts -μgs/ml- of NPs) the NPs dispersions (5 ml) were dialyzed against vigorously stirred deionized water (250 ml) for 1 min using dialysis tubing (molecular weight cutoff = 14 kDa) to remove EDC-urea byproduct and directly used for DLS. This should minimize any potential artifacts in NPs sizes or charges due separation methods (e.g., lyophilization or centrifugation) (Zhang et al., 2018) other than the influence of studied variables, i.e., pH, Ca^2+^, and FBS. (2) For dissolution, *in vitro* release studies, and cell lines cytotoxicity studies (which require significantly more amounts of NPs – *ca*. mg/ml): Blank and loaded NPs were separated from the dispersion by centrifugation at 4,000 rpm for 45 min, washed gently by distilled water and lyophilized using Operon Freeze-Dryer - (Korea) at vacuum pressure of 0.05 mbar. The condenser surface was maintained at 55°C. Lyophilized samples were stored in light-protected containers at −20°C for later use.

### Characterization of Semi-synthetic Polymers and Corresponding NPs

Infrared spectrums (Fourier-transform Infrared-FTIR or Attenuated Total Reflection-ATR) were collected using Shimadzu-FTIR-8400S (Japan) and Thermo DS spectrometer (Germany). Ionotropically- or covalently- crosslinked NPs for infrared analysis were prepared as were previously described. However, the resulting NPs dispersion was precipitated using acetone (150 ml), washed three times with absolute ethanol (100 ml), and finally with acetone (100 ml) then dried overnight at 35°C.

Crosslinked matrices (ionotropically- or covalently-crosslinked) or polymer samples (chitosan and semi-synthetic derivatives) were crushed using mortar and pestle and mixed with potassium bromide at 1:100 ratios and compressed to a 2 mm semitransparent disk over 2 min for FTIR analysis. For ATR analysis, the powdered samples were placed directly into the diamond crystal of the instrument. The spectrums were recorded over wavelength range of 4,000–400 cm^−1^.

Thermal analysis using Differential Scanning Calorimetry (DSC) using a DSC 823^e^ Mettler Toledo (Thermo Electron Scientific Instruments Corp., Madison, WI). Samples were prepared by weighing (3–7 mg) of each polymer in aluminum sample pans and sealing them using the Toledo sample encapsulation press. Each sample was heated from 25 to 350°C at 10°C/min heating rate under N_2_ purge using an empty sealed pan as a reference. Calibration with the standard (indium) was undertaken prior to subjecting the samples for study.

Although acetone precipitation is rather drastic method vis-à-vis NP sizes and charges, it is harmless to NPs properties monitored by IR and DSC, namely, covalent and strong reversible interactions within NPs polymeric matrices. Moreover, acetone precipitation yields large enough NPs amounts suitable for IR and DSC studies.

### NPs Size Analysis, Surface Charge Measurement, and Stability Studies Under Variable pH, CaCl_2_ Conditions and 10% FBS Solution

Aliquots of crosslinked NPs (ionotropic or covalent) dispersions (2 ml) were evaluated by dynamic light scattering (DLS) either directly (at preparation pH), or after being subjected to variable pH conditions (1.2, 6.8, 7.4, and 12.0) CaCl_2_ concentrations (0.1, 0.2, 0.3, 0.4, and 0.5 M), or fetal bovine serum (10% FBS in PBS at 7.4 pH). pH adjustments were achieved by aqueous NaOH (0.1 M) or HCl (1.0 M) and monitored using pH-meter (Trans Instruments, Singapore). Each sample was vigorously stirred for 1 min at room temperature to ensure homogenous dispersion and was then macroscopically inspected for haziness (Tyndall effect) or aggregate formation. Only samples with hazy appearance were analyzed by DLS while those showing aggregates were discarded. Samples were evaluated by DLS after 2 h exposure to variable pH, CaCl_2_ concentrations, or 10% FBS solution. Particle size, polydispersity index (PDI), and zeta potential were calculated by determining the electrophoretic mobility of NPs dispersions followed by applying the Stokes-Einstein and Henry equations. The following parameters were assumed in the calculations: Media viscosity = 0.8872 cP, dielectric constant = 78.5, temperature = 25°C. The measurements were performed using Zetasizer Nano ZS (4.0 mW He-Ne laser, 632.8 nm, Malvern Instruments, UK) while the respective calculations were performed using Zetasizer software version 7.11. The measurements were done in triplicates at 25°C and the average size and zeta potential were recorded.

The morphological characteristics of NPs were studied by transmission electron microscope (TEM) (Morgagni (TM) FEI 268, Holland) using Mega-View Camera. The samples were immobilized on copper grids for 10 min and dried at room temperature prior to investigation by TEM.

### Loading Capacities and *in vitro* Release Studies

To aqueous solutions of the particular chitosan or chitosan derivative (0.1% w/v, 100 ml) in HCl (4.8 mM) MB or DOX (10 or 100 mg to prepare 1:10 or 1:1 polymer to drug ratios, respectively) were added and stirred for 30 min. The resulting solutions were separated into (5 ml) fractions and the appropriate crosslinker (0.1%w/v HMP, 0.2%v/v PPA, or 0.4% w/v TPP) was added dropwise to selected fractions until the development of hazy dispersions. Then, EDC (25 mg) was added to each fraction for covalent crosslinking. The reaction mixtures were stirred vigorously over 1 min and allowed to stand over 1 h. The reactions were terminated by centrifugation (Megafuge 8R, Thermo Scientific-Slovenia) at 4,000 rpm for 45 min at 4°C, then NPs pellets were retained and the supernatant discarded. NPs pellets were gently washed with deionized water and placed overnight in deep freezer (−80°C, Polar 530 V, Italy) then lyophilized as mentioned earlier. Lyophilized samples were stored in light-protected containers at −20°C for later use (stable over 8-months period).

The release profiles and loading capacities of loaded NPs were determined using the dialysis bag method. An exactly weighed amounts of drug-loaded lyophilized NPs were re-dispersed in HCl (3 ml, 4.8 mM) in a dialysis sac and was subsequently put in an amber-glassed bottle containing TRIS base buffer (17 ml, pH 7.4). The assembly was placed in a shaking incubator (DAIKI -Scientific Co, Korea) at 100 rpm and 37°C. Samples (2 ml) were withdrawn from TRIS buffer at specified time intervals and immediately replaced with an equivalent volume of fresh buffer. For MB quantification, samples absorbances were measured using UV-Visible spectrophotometer (Thermo Fisher Scientific model B40-210600, China) at λ_max_ = 666 nm. For DOX, samples were measured using Shimadzu spectrofluorometer (model RF-5301PC, Japan) at excitation wavelength λ_max_ 485 and emission λ_max_ 558 nm. Slit widths were adjusted to 5 for excitation and emission. Unloaded NPs were used as blanks.

The released amounts were calculated from properly drawn calibration curves. The release profiles were repeated in triplicates and expressed as average cumulative amounts of released drug per mg NPs. The standard deviation (SD) was used as variability descriptor.

To determine the amounts of loaded drugs (i.e., MB or DOX) in covalently crosslinked NPs: The cumulative amounts of each drug released over 24 h upon dissolution (see above) were added to amounts released upon degrading the respective loaded NPs (core loading). NPs degradation was performed as follows: Remaining nanoparticles within dialysis bags (after drug releasing studies) were collected by centrifugation at 4,000 RPM over 10 min and washed gently with distilled water, then suspended in HCl (2.0 M) at 72°C for 3 h in case of MB loaded NPs and ultrasonicated using ultrasonic processor (Cole-Parmer, USA) for DOX loaded NPs (at 50% amplitude for 10 min) (Tang et al., 2003).

The released amounts were calculated from properly drawn calibration curves (Cabrera and Van Cutsem, 2005). The loading capacity is calculated as in the following equation:

$$\begin{array}{l} Loading\;Capacity=\frac{Amount\;of\;drug\;in\;mg\;of\;NPs}{Weight\;of\;NPs\left(mg\right)}\times 100\% \end{array}$$

### Cytotoxicity Studies

The cytotoxicity of (drug-free or DOX loaded NPs) was performed using the CellTiter Non-Radioactive Cell Proliferation Assay Kit® (Promega, USA). Free DOX was used as a positive control. A stock solution of 50 μM free DOX or its equivalent amount of DOX- loaded NPs was used to prepare serial dilutions from 0.05 to 50 μM in fresh media. The culture of MCF-7 cell line was maintained in RPMI 1640 medium, supplemented with 10% (v/v) heat-inactivated fetal bovine serum (FBS), 2 mM l-glutamine, 100 U/ml and 100 μg/ml penicillin–streptomycin (EURO Clone, Italy). The cells were trypsinized by trypsin-EDTA (EURO Clone, Italy) and centrifuged to form a pellet of the cells. The supernatant was discarded. The cell pellet was then re-suspended in its growth medium. The cell stock was diluted to the desired concentration (7 × 10^4^ cells/ml). The cell suspension was transferred to 96 well-plates by adding 100 μl of the cell suspension to each well. The plates were incubated in a humidified atmosphere at 37°C and 5% CO_2_ for 24 h to allow the cells to be in their exponential growth phase at the time that NPs suspension or free DOX were added. It is important to mention that ultrasonication (30% amplitude for 2 min) was used to find fine NPs suspension and the same condition was applied for free DOX. The spent medium (deprived of nutrients) was discarded and replaced by fresh medium with an appropriate concentration of the NPs suspension or free DOX. After 72 h of incubation, MTT assay solution was added. The plates were incubated for 4 h in the absence of light at 37°C then the stop solution was added. The number of live cells was identified after 30 min of stop solution addition by measuring the absorbance at 570 nm using a 96-well plate reader (BioTek Instruments, U.S.A). The following equation was used to calculate cell viability.

$$\begin{array}{l} {}[Cell\;viability\%\\ =\;\frac{Absorbance\;\left(570\;nm\right)\;of\;Doxorubicin\;treated\;sample}{Absorbance\;\left(570\;nm\right)\;of\;control\;sample}\times 100\%] \end{array}$$

The results of the MTT cell proliferation assay were analyzed using excel. The inhibitory concentration (IC_50_) values, which are the drug concentration at which 50% of cells are viable, were calculated from the logarithmic trend line of the cytotoxicity graph. The cellular morphological changes related to NPs-induced cytotoxicities were monitored using inverted light microscope (Vert. A1, AX10, Carl Ziess, Germany) of MCF-7 cells after exposure to DOX-loaded NPs and free DOX (10.0 μM) over 72 h incubation. Unloaded NPs and untreated cells were used as controls.

#### Assessment of NPs Cellular Uptake Using Confocal Microscopy

MCF-7 cells were seeded onto poly-L-lysine coated round coverslips (prepared by incubation in poly-L-lysine aqueous solution (0.01% w/v) over 1 h at room temperature) in a 12-well plate at 5 × 10^4^ cells/well in RPMI culture medium and left over 24 h. DOX-loaded NPs or free DOX (1.0 μM), suspended in tissue culture media, were directly applied to coverslips adhered cells and incubated over 4 h at 37°C Subsequently, the culture media were removed and wells were washed two times with PBS. Cells were then fixed by paraformaldehyde solution (4%) at room temperature over 20 min then washed two times with PBS. Subsequently, triton-x solution (0.5% v/v) was added to wells and incubated for 10 min then washed two times with PBS. Thereafter, the coverslips were removed and slowly flipped over clean glass slides covered with 50.0 μL DAPI stain (Prolong^TM^ Diamond Antifade Mountant with DAPI) and left overnight at room temperature under dark conditions. Fixed cells were imaged at laser/detector wavelengths of 488 nm/614–742 nm for DOX and of 405 nm/410–585 nm for DAPI using confocal laser scanning microscope (LSM 780, Carl Ziess, Germany) by 63×/1.4 oil lens. Untreated cells (i.e., with DOX-loaded NPs or free DOX) were assessed as controls. NPs uptake was also evaluated using wide-field fluorescence microscopy (Axio Imager Z2, Carl Ziess, Germany).

### Statistical Analysis

All experimental data were presented as mean and standard deviation (SD). Microsoft Excel Software 2007 (Microsoft Corp., Redmont, WA, USA) was used to calculate means, standard deviations of the size, zeta potential, loading and cumulative amount released, and to create graphs. Excel was also used to calculate *t*-test and *p-values*. Microscopic images were labeled using ZEN software (version 2012, SP5).

## Results and Discussion

### Synthesis of Chitosan-Dicarboxylic Acid Conjugates

Chitosan-phthalate (CP) and chitosan-phenylsuccinic (CPS) were recently reported to yield TPP-based NPs of optimal properties for drug delivery (Dmour and Taha, 2017) prompting us to select them for our current NPs and drug release studies.

CP and CPS were synthesized by the reaction of chitosan with phthalic or phenylsuccinic anhydride in neutral pH. Catalytic amount of pyridine was added to push the anhydride/amine acylation chemistry (Aiedeh and Taha, 1999; Dmour and Taha, 2017). Figure 1G summarizes the conjugation reactions. The resulting polymers were characterized by NMR spectroscopy. Figure S29 shows the ^1^H NMR spectra of the grafted polymers.

### NPs Formulation

**Figure 2:**
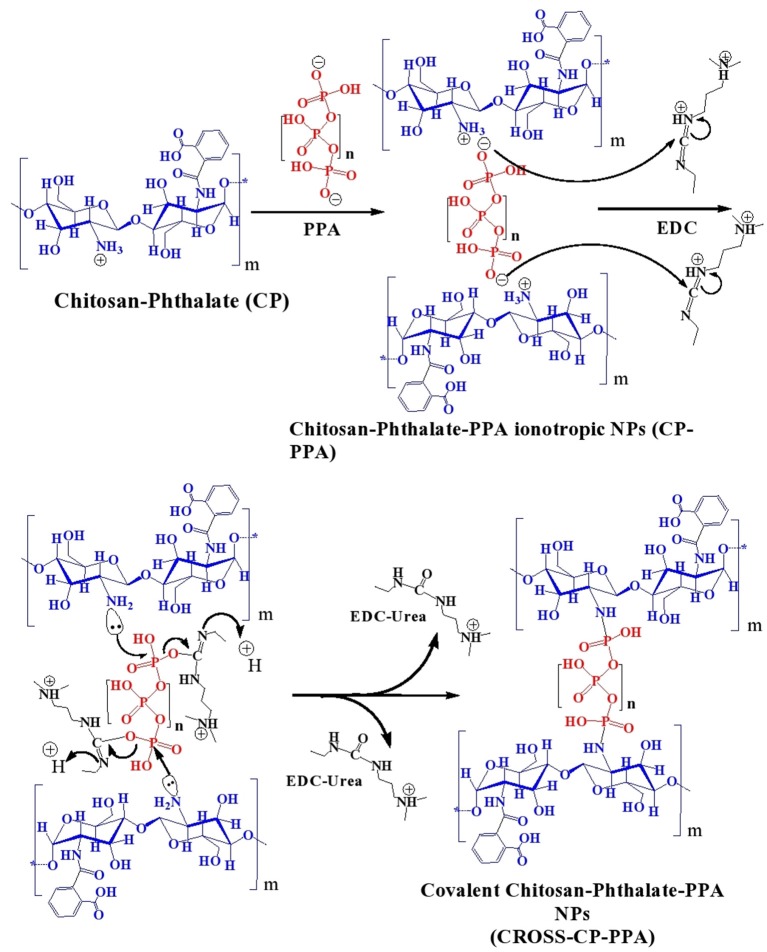
Tandem ionotropic/covalent crosslinking of CP with PPA and EDC.

Initially, chitosan and conjugation derivatives (CP and CPS) were used to prepare NPs by ionotropic gelation with three polyphosphates crosslinkers, namely TPP, PPA, and HMP. Table 1 summarizes the prepared NPs and their abbreviated names. Figure 2 summarizes the formulation of CP-PPA NPs as example.

**Table 1:** Prepared ionotropically-crosslinked chitosan NPs, their corresponding crosslinkers, and abbreviated names.

**Polymer**		**Ionotropic NPs**	
**Derivative**	**Abbreviation**	**Cross-linker**	**Abbreviation**
Unmodified chitosan	C	Tripolyphosphate	C-TPP
		Polyphosphoric acid	C-PPA
		Hexametaphosphate	C-HMP
Chitosan phthalate	CP	Tripolyphosphate	CP-TPP
		Polyphosphoric acid	CP-PPA
		Hexametaphosphate	CP-HMP
Chitosan phenylsuccinate	CPS	Tripolyphosphate	CPS-TPP
		Polyphosphoric acid	CPS-PPA
		Hexametaphosphate	CPS-HMP

Ionotropic gelation proceeds via attraction between protonated amine groups of chitosan (or chitosan's derivatives) and phosphate anions of TPP, HMP, or PPA. These interactions weaken the surface charge of chitosan and therefore reduce chitosan's aqueous solubility leading to spontaneous NPs formation (Figure 2). Ionotropic crosslinking was practically performed by titrating solutions of chitosan (or its derivatives, pH values as in Table 2) with aqueous polyphosphate crosslinkers until the appearance of hazy (opalescent) dispersions. Chitosan conjugates (i.e., CP and CPS) tended to consume significantly lesser amounts of PPA and HMP crosslinkers to form NPs compared to unmodified chitosan, as in Table 2. However, this trend is not observed with TPP, i.e., unmodified chitosan and chitosan conjugates required the same levels of TPP to form ionotropic NPs. We believe this trend is because conjugated chitosans fold in acidic aqueous conditions in such way to keep hydrophobic acidic conjugates (unionized phthalic and phenylsuccinic acids under acidic NPs preparation conditions) confined within the interior of newly formed NPs. Apparently, TPP acts at NPs surfaces (i.e., surface crosslinker) and thus being far from core carboxylic acid-substituted amines, the amount of TPP needed for NPs formation is independent of chitosan conjugation. In comparison, HMP (and to a lesser extent PPA) seem to act as core crosslinker in direct proximity to conjugated amines (i.e., with phthalic and phenylsuccinic acids) within newly formed NPs cores, such that carboxylic acid conjugation reduces the number of core cationic amines exposed to HMP with the concomitant reduction in the required HMP phosphate counter-ions necessary to weaken chitosan's charge leading to spontaneous NPs formation. This conclusion is supported by the significantly higher positive surface charges of HMP-based NPs compared to TPP-based NPs (see section NPs Behavior under Variable pH/Calcium Ion Conditions, NPs Sizes and Surface Charges and **Table 4**).

**Table 2:** Crosslinking conditions of ionotropic and covalent NPs together with their corresponding abbreviated names.

**Ionotropic NPs**	**Amount of ionotropic Crosslinker (mg) per polymer (mg)^**a**^**	**pH of**			**Abbreviations of covalent NPs**
		**Polymeric dispersion^**b**^**	**Ionotropic NPs dispersions^**b**^**	**NPs dispersions after EDC addition^**b**^**	
C-TPP	0.35 ± 0.05	4.32 ± 0.05	5.53 ± 0.03	5.59 ± 0.34	—^c^
C-PPA	0.20 ± 0.07		2.04 ± 0.01	2.05 ± 0.03	—^c^
C-HMP	0.16 ± 0.04		4.69 ± 0.17	4.95 ± 0.16	—^c^
CP-TPP	0.38 ± 0.02	2.56 ± 0.04	3.00 ± 0.03	5.18 ± 0.19	CROSS-CP-TPP
CP-PPA	0.09 ± 0.02		1.87 ± 0.012	3.14 ± 0.05	CROSS-CP-PPA
CP-HMP	0.10 ± 0.03		2.62 ± 0.15	2.80 ± 0.075	—^c^
CPS-TPP	0.32 ± 0.02	2.54 ± 0.04	3.55 ± 0.27	3.79 ± 0.015	—^c^
CPS-PPA	0.09 ± 0.01		2.08 ± 0.06	2.12 ± 0.078	—^c^
CPS-HMP	0.06 ± 0.02		2.55 ± 0.01	3.08 ± 0.15	CROSS-CPS-HMP

a *Amounts of phosphate crosslinkers necessary for optimal mono-dispersed ionotropic NPs. Lesser or greater amounts lead to either loss of NPs, larger particles, or aggregates*.

b *Means were recorded for triplicate measurements ± standard deviation*.

c *Addition of EDC failed to produce stable NPs under variable pH and CaCl_2_ conditions*.

Another interesting observation in Table 2 is related to the change in pH profiles of chitosan dispersions upon grafting to phthalic and phenylsuccinic acids. Clearly from Table 2, the pH of the polymeric dispersions became more acidic upon conjugation to dicarboxylic acids, which is not surprising due to the fact conjugation consumes basic amines groups within chitosan and converts them into neutral amidic linkages. However, pH shifts accompanying conjugation to phthalic and phenylsuccinic acids (CP and CPS, respectively) were identical (from pH 4.32 to pH 2.5) suggesting identical substitution degrees in both cases (CP and CPS). In fact, we could calculate the degrees of phthalic and phenylsuccinic acids substitution on chitosan to be 43%. The calculation is based on subtracting hydrogen ion concentration before and after dissolving each chitosan derivative in certain predetermined volume of HCl (4.8 mM).

EDC was added to the generated ionotropic NPs for covalent crosslinking (Figure 2). Acidic conditions are necessary to protonate EDC's imino-nitrogen atoms and enhance its reactivity. The coupling chemistry was performed by excess EDC to ensure reaction completion, particularly under the sterically hindering environment of the polymer. Both EDC and EDC-urea byproduct are water soluble and allow easy subsequent polymer purification by dialysis in aqueous conditions.

Table 2 shows another interesting observation: Successful EDC-mediated covalent crosslinking (i.e., CP-TPP, CP-PPA, and CPS-HMP) significantly shifted the pH of corresponding NPs dispersions toward more basic values, while those that failed EDC crosslinking maintained the same pH values prior to EDC addition. In fact, success of EDC crosslinking can be easily monitored by observing the pH shifts of corresponding NPs dispersions upon adding EDC. Transition of EDC to EDC-urea (upon covalent crosslinking) consumes acidic protons causing the observed basic shifts, as in Figure 2. Failure of the conjugation reaction means EDC fails to convert into EDC-urea and thus fails to abstract protons from the medium (as in the cases of C-TPP, C-PPA, C-HMP, CP-HMP, CPS-TPP, and CPS-PPA NPs, Table 2). Subsequent probing with infrared, thermal and NPs stability profiles (see next) unequivocally supported our conclusions, i.e., success of EDC-induced covalent crosslinking in CP-TPP, CP-PPA, and CPS-HMP NPs cases (via forming phosphoramide crosslinks) and failure of covalent crosslinking in C-TPP, C-PPA, C-HMP, CP-HMP, CPS-TPP, and CPS-PPA NPs cases (see section NPs Behavior under variable pH/calcium ion conditions, NPs sizes and surface charges).

### Characterization of Polymeric Intermediates and NPs

#### Infrared Spectroscopy (IR)

**Figure 3:**
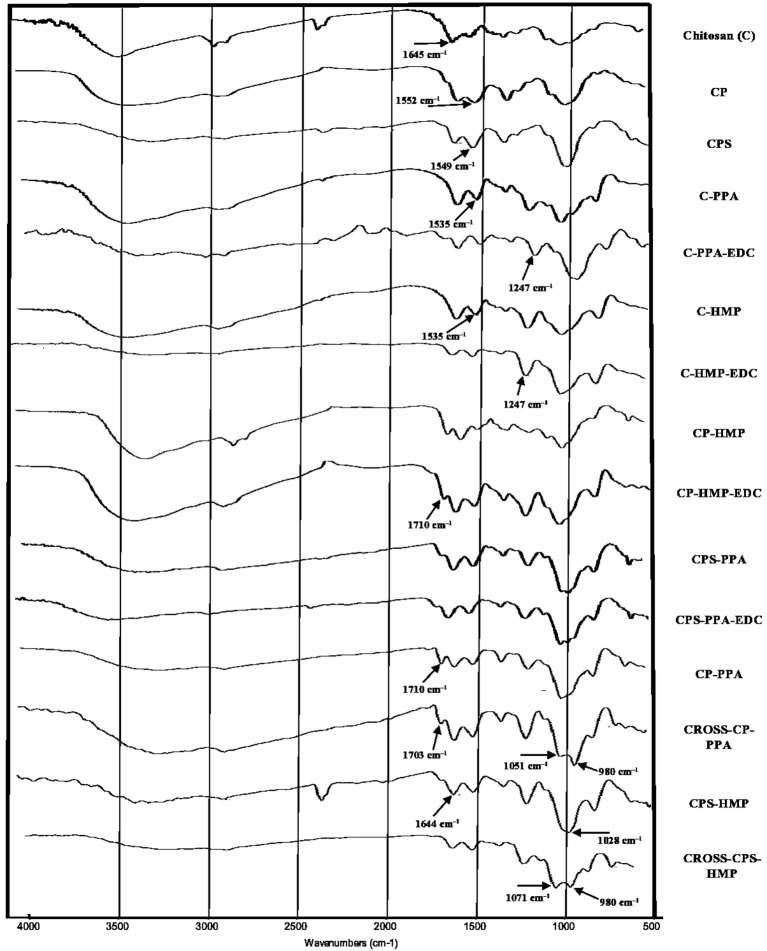
IR spectrums of unmodified parent chitosan, corresponding derivatives, and NPs (ionotropically and covalently crosslinked). Individual infrared spectrums are shown in Figures S2–S16.

To probe chemical conjugation of chitosan and subsequent NPs formation, we opted to use IR and differential scanning calorimetry (DSC). Figure 3 shows the infrared spectra of parent chitosan, corresponding derivatives, and selected NPs (ionotropically and covalently crosslinked). As in Figure 3, chitosan's IR spectrum exhibits NH/OH stretching and N-H bending vibrations (at 3,400 and 1,645 cm^−1^, respectively) (Brugnerotto et al., 2001). However, it lacks stretching amide I carbonyl band at 1,655 cm^−1^ indicating considerable deacetylation (Khan et al., 2002; de Alvarenga, 2011), which agrees with the deacetylation degree reported by the manufacturing company (*ca*. 85%, Sigma Aldrich, USA).

Grafting chitosan with carboxylic acid anhydrides was evidenced in the corresponding infrared spectra by new stretching bands within 1,549–1,553 cm^−1^ range (clear in CPS and CP spectra in Figure 3) corresponding to carboxylate and amide II stretching vibrations accompanying the conjugation to phthalic and phenylsuccinic acids. It's noteworthy to mention that amide I stretching bands seem to be concealed by N-H bending vibrations of remaining chitosan amines at 1,640 cm^−1^.

Although IR is blind to electrostatic interactions, and therefore, is not able to probe ionotropic phosphate-ammonium interactions, the acidic pH required for ionotropic gelling (Table 2) protonated amine and carboxylate residues of the polymers causing considerable change in the respective infrared spectra: In unmodified chitosan (C, Figure 3), acidification and treatment with polyphosphate crosslinkers lead to appearance of a new band at 1,535 cm^−1^ in C-PPA and C-HMP NPs, corresponding to bending vibrations of ammonium groups, alongside the original band at 1,641 cm^−1^ which correspond to bending vibrations of the amine groups. Conversely, acidification/phosphate treatment of anhydride-grafted chitosan derivatives significantly protonated the carboxylate residues into carboxylic acids with the concomitant emergence of new shoulder bands in CPS-HMP NPs and CP-PPA NPs spectra at *ca*. 1,710 cm^−1^ related to carboxylic acid carbonyl stretching. Additionally, ionotropically-crosslinked NPs exhibited new distinct band at 1,247 cm^−1^ corresponding to P=O stretching vibrations of the phosphate crosslinkers (Nyquist et al., 1967; Nishi et al., 1986; Dmour and Taha, 2017).

Infrared spectroscopy was also used to investigate covalent crosslinking reactions resulting from treating ionotropic NPs with EDC. From Figure 3, treating CP-PPA, and CPS-HMP NPs with EDC (i.e., to yield CROSS-CP-PPA, and CROSS-CPS-HMP, respectively) was accompanied by new significant band at *ca*. 980 cm^−1^ corresponding phosphoramide bond formation within NPs (Nyquist et al., 1967; Nishi et al., 1986; Dmour and Taha, 2017). This band is absent from infrared spectrums of NPs that failed covalent crosslinking despite exposure to EDC, e.g., C-PPA-EDC, C-HMP-EDC, CP-HMP-EDC, and CPS-PPA-EDC, as in Figure 3.

Intriguingly, carboxylic acid bands seen upon acidification/polyphosphate crosslinking (C=O stretching band at ≈ 1,710 cm^−1^ seen in CP-PPA and CPS-HMP) remained after treatment with EDC in CROSS-CP-PPA as well as in TPP-based NPs (Dmour and Taha, 2017), which indicate that the polymeric carboxylic acid moieties were not involved (or minimally involved) in covalent crosslinking within NPs matrices. However, this band (i.e., at 1,710 cm^−1^) disappeared totally in CROSS-CPS-HMP NPs spectrum despite acidic conditions (pH 3.08, which should protonate remaining carboxylates into carboxylic acids). This suggests that carboxylic acid moieties in CROSS-CPS-HMP NPs were consumed in EDC-mediated amide bond forming reaction additional to the phosphoramide formation reaction mentioned earlier. We believe this extra-crosslinking reaction is due to the fact that HMP is primarily core-crosslinking agent (see section NPs behavior under variable pH/calcium ion conditions, NPs sizes and surface charges) with little abundance at the outer NPs surface, thus leaving the chance for slower amide forming crosslinking (coupling free carboxylic acids of grafted anhydrides with chitosan amines) to take place at the NPs surfaces.

It remains to be mentioned that grafted carboxylic acid moieties are essential for successful formation of phosphoramide bonds as they catalyze coupling of polyphosphates with polymeric amine groups (Dmour and Taha, 2017). This explains EDC failure to achieve phosphoramide bonds in the lack of grafted carboxylic acid groups, as seen in the IR spectra of EDC-treated C-PPA and C-HMP NPs (absence of phosphoramide bands at *ca*. 980 cm^−1^ as in Figure 3). Strangely, however, all our attempts to crosslink chitosan (unmodified) NPs with polyphosphates (TPP, HMP, or PPA) in acetic acid were futile, suggesting that the carboxylic acid catalyst need to be covalently attached to NPs matrix to catalyze the EDC coupling chemistry successfully.

#### Thermal Analysis

**Figure 4:**
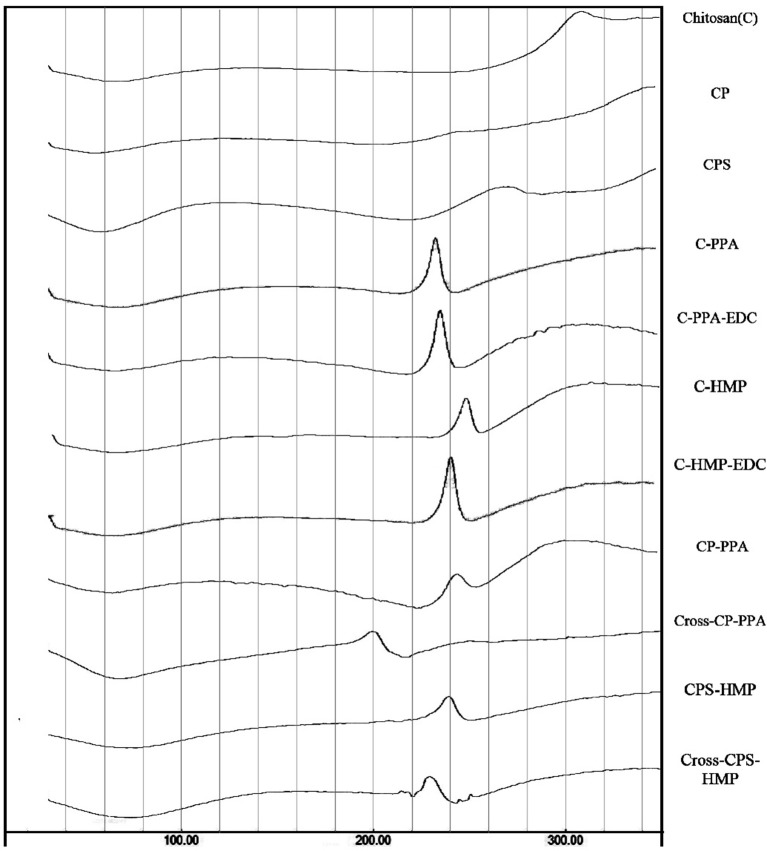
DSC thermograms for unmodified chitosan (C), chitosan phthalate (CP), chitosan phenylsuccinate (CPS), and their ionotropic/covalent corresponded nanoparticles. The individual thermal DSC traits are shown in Figures S17–S27.

DSC thermograms of chitosan, carboxylic acid derivatives, and NPs (both ionotropic and covalent) are shown in Figure 4. Chitosan shows typical polysaccharide thermal trait characterized with two bands. The first is endothermic wide band that extends from 40° to 100°C corresponding to polymeric dehydration. The second thermal event is exothermic band extending from 280 to 320°C corresponding to polymeric degradation. The thermal trait of CP is rather flat, while CPS shows shallow exothermic band extending from *ca*. 220–274°C probably linked to thermally-mediated amide forming reaction linking phenylsuccinic acid moieties and adjacent chitosan's amine groups in CPS. Similar exothermic feature was evidenced upon attaching phthalic anhydride to chitosan-lactate (Al Bakain et al., 2015). Probing the thermal characteristics of ionotropic NPs demonstrates intriguing exothermic peaks (extending from 225° to 247°C for CP-PPA NPs and from 220° to 240°C in CPS-HMP NPs) resulting from certain heating-induced exothermic incident within NP matrices. The most likely explanation for these peaks is heat-induced phosphoramide forming reaction linking chitosan's amine groups with polyphosphate crosslinkers (see Figure S1) (Dmour and Taha, 2017). We excluded the prospect that these peaks are due to amide bond formation between carboxylic acids (phenylsuccinic or phthalic acids) and chitosan's aminosugar monomers because the same peaks emerged in the thermograms of C-PPA and C-HMP NPs (both are based on unmodified chitosan), as in Figure 4.

EDC coupling was also manifested in the DSC traits. EDC coupling weakened the exothermic peaks and moved them to lower temperatures (from 245 to 200°C in CP-PPA and from 240 to 230°C in CPS-HMP). The most logical reason for the observed EDC-induced decrease of exothermic bands is that EDC crosslinking depleted a considerable fraction of phosphate crosslinker molecules, and consequently, eliminated them from the thermally-induced reaction with aminosugar monomers of chitosan (or acid-grafted chitosan derivatives). Accordingly, the fact that CROSS-CP-PPA exhibited the most drastic attenuation in the exothermic peak implies that EDC crosslinking was most efficient in this case. In contrast, the DSC traits of C-PPA and C-HMP remained unchanged after EDC addition (i.e., C-PPA-EDC and C-HMP-EDC, in Figure 4) which further supports the notion that the presence of conjugated carboxylic acids is essential for EDC mediated formation of phosphoramide covalent bonds.

#### NPs Behavior Under Variable pH/Calcium Ion Conditions, NPs Sizes and Surface Charges

**Figure 5:**
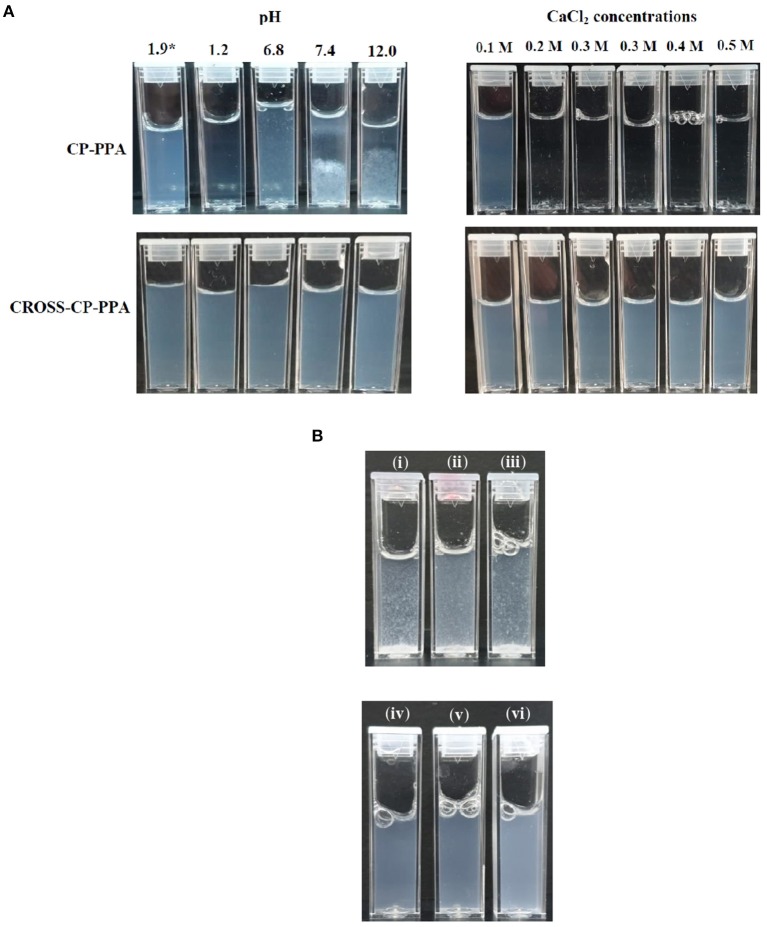
Stabilities of ionotropic and covalent nanoparticles in variable pH, calcium chloride, and serum conditions. **(A)** Responses of CP-PPA and CROSS-CP-PPA NPs to variable pH (left) and CaCl2 conditions (right). *pH 1.9 corresponds to preparation pH. **(B)** Stabilities of ionotropic (up) and covalent (down) NPs in 10% FBS solution. (i) CP-TPP, (ii) CP-PPA, (iii) CPS-HMP, (iv) CROSS-CP-TPP, (v) CROSS-CP-PPA, and (vi) CROSS-CPS-HMP.

Table 3 summarizes NPs size information and how they behave under variable pH, calcium ions concentrations, and 10% FBS. Figure 5A shows how CP-PPA and CROSS-CP-PPA NPs respond to variable pH, and calcium chloride conditions, while Figure 5B shows how CP-TPP, CP-PPA, CPS-HMP NPs dispersions and their corresponding covalently crosslinking analogs behave in 10% FBS solution. Ionotropic NPs were stable over pH range of 2.0–6.0. However, they immediately (within seconds) dissolved in acidic pH (1.2) to form clear solutions, while at pH values ≥ 6.8, with or without FBS, they created macroscopical aggregates, as seen in Figures 5A,B.

**Table 3:** Size properties of ionotropic and covalent NPs under varying pH, calcium chloride conditions, and 10% FBS solution.

		**Ionotropic NPs**	**NPs After EDC addition**										
**NPs**	**NP Property^**a**^**	**At Preparation Conditions**	**pH**					**CaCl**_****2****_ **(M)**^**f**^					
				**1.2^**f**^**	**6.8^**f**^**	**7.4**		**12.0^**f**^**	**0.1**	**0.2**	**0.3**	**0.4**	**0.5**
						**Aqueous conditions**	**FBS (10%) v/v)**						
C-TPP	Size (nm)	205.4 ± 3.8	—^c^	Clear	Aggregate	Aggregate	—^e^	Aggregate	Clear	Clear	Clear	Clear	Clear
	PDI^b^	0.42 ± 0.02	—^c^	—	—	—	—^e^		—	—	—	—	—
C-PPA	Size (nm)	118.0 ± 2.4	—^c^	Clear	Aggregate	Aggregate	—^e^	Aggregate	Clear	Clear	Clear	Clear	Clear
	PDI^b^	0.35 ± 0.03	—^c^	—	—	—	—^e^		—	—	—	—	—
C-HMP	Size (nm)	458.8 ± 21.5	—^c^	933 ± 151^d^	Aggregate	Aggregate	—^e^	Aggregate	Clear	Clear	Clear	Clear	Clear
	PDI^b^	0.52 ± 0.11	—^c^	0.55 ± 0.1^d^	—	—	—^e^		—	—	—	—	—
CP-TPP	Size (nm)	148.3 ± 11.2	159.8 ± 10.3	195.9 ± 5.4	249.9 ± 6.7	319.4 ± 40.3	173.5 ± 3.71	334.9 ± 8.7	230.4 ± 4.4	269.5 ± 11.6	232.7 ± 31.3	234.6 ± 4.7	256.8 ± 11.2
	PDI^b^	0.16 ± 0.02	0.16 ± 0.18	0.17 ± 0.02	0.26 ± 0.03	0.35 ± 0.05	0.31 ± 0.05	0.35 ± 0.7	0.21 ± 0.01	0.25 ± 0.03	0.19 ± 0.028	0.19 ± 0.02	0.23 ± 0.01
CP-PPA	Size (nm)	133.5 ± 8.0	158.1 ± 6.5	189.5 ± 10.0	171.3 ± 12.2	199.1 ± 2.7	263.8 ± 12.8	228.9 ± 6.0	193.6 ± 4.0	274.5 ± 19.8	374.5 ± 30.7	260.8 ± 34.5	471.6 ± 26.4
	PDI^b^	0.28 ± 0.04	0.31 ± 0.04	0.29 ± 0.02	0.23 ± 0.01	0.29 ± 0.04	0.44 ± 0.05	0.30 ± 0.03	0.19 ± 0.03	0.29 ± 0.07	0.41 ± 0.06	0.29. ± 0.07	0.29 ± 0.05
CP-HMP	Size (nm)	415.4 ± 16.7	—^c^	259.4 ± 10.5^*d*^	Aggregate	Aggregate	—^c^	Aggregate	Clear	Clear	Clear	Clear	Clear
	PDI^b^	0.43 ± 0.06	—^c^	0.37 ± 0.05^*d*^	—	—	— ^c^	—	—	—	—	—	—
CPS-TPP	Size (nm)	156.6 ± 3.3	—^c^	Clear	Aggregate	Aggregate	— ^c^	Aggregate	Clear	Clear	Clear	Clear	Clear
	PDI^b^	0.09 ± 0.01	—^c^	—	—	—	— ^c^	—	—	—	—	—	—
CPS-PPA	Size (nm)	120.6 ± 3.3	—^c^	Clear	Aggregate	Aggregate	— ^c^	Aggregate	Clear	Clear	Clear	Clear	Clear
	PDI^b^	0.23 ± 0.01	—^c^	—	—	—	—^c^	—	—	—	—	—	—
CPS-HMP	Size (nm)	331.7 ± 10	254.0 ± 30.0	299.9 ± 13.0	200.1 ± 4.0	258.2 ± 44	335 ± 49.2	349.8 ± 39	283.8 ± 8.0	265.5 ± 10.0	247.1 ± 5.8	248.6 ± 7.6	254.8 ± 11.0
	PDI^b^	0.39 ± 0.03	0.26 ± 0.02	0.27 ± 0.01	0.26 ± 0.03	0.38 ± 0.09	0.58 ± 0.10	0.49 ± 0.09	0.29 ± 0.03	0.31 ± 0.03	0.33 ± 0.04	0.34 ± 0.05	0.30 ± 0.04

a *Each value represents the average of triplicate measurements ± standard deviation. DLS graphs showing the average sizes of example NPs are shown in Figure S28*.

b *Polydispersity index*.

c *Covalent crosslinking failed (EDC failed to covalently crosslink NPs based on infrared and DSC evidence, see text)*.

d *Shown data are for ionotropic NPs (as EDC failed to covalently crosslink NPs based on infrared and DSC evidence, see text)*.

e *Not tested*.

f *Aqueous conditions (no FBS)*.

Acidic conditions hydrolyze polyphosphate crosslinkers (Lind, 1948) and impair their abilities to electrostatically attract chitosan's ammonium moieties leading to observed dissolution of NPs. However, although all ionotropic NPs formulas lost their integrities upon exposure to acidic pH (1.2), C-HMP and CPS-HMP NPs retained their integrities under such conditions, as in Table 3. Resistance of HMP-based ionotropic NPs to drastic acidic pH conforms to our proposition that HMP is core crosslinker and stays within the confinement of NPs cores protected from hydrolysis by the external acidic solution.

Conversely, basic conditions deprotonate chitosan's ammonium residues resulting in loss of significant fraction of chitosan's positive charge, thus undermining its ability to electrostatically interact with negatively charged phosphates crosslinkers. Additionally, the positive surfaces of chitosan NPs serve as deflocculants adding further stability to the NPs dispersion. Losing these charges flocculates NPs suspension and forces them to aggregate into macroscopical particulate clusters.

Similarly, Table 3 and Figure 5 show ionotropic NPs to dissolve completely and immediately in CaCl_2_ solutions regardless of concentration. Calcium ions form stable chelates with phosphate ions (Rehfeld et al., 1977) and therefore sequester phosphate from being electrostatically complexed to chitosan. This leads to complete dissolution of ionotropic NPs under the influence of calcium ions as in Table 3 and Figure 5A.

The most notable observation in Table 3 and Figure 5 is that covalently crosslinked NPs maintained their opalescent appearance, nano-sizes and resisted extreme pH environment, FBS conditions, and increasing CaCl_2_ levels over at least 2 h exposure periods. This is not unexpected because covalent crosslinking decouples the stabilities of crosslinked polymeric matrices from solution pH or calcium ions. We reported similar findings for TPP-covalently-crosslinked NPs based on modified chitosan (Dmour and Taha, 2017).

Interestingly, however, EDC failed to covalently crosslink CP-HMP, CPS-PPA, and CPS-TPP NPs as evident from their total lack of stability under variable pH and CaCl_2_ conditions (and lack of phosphoramide IR stretching vibrations in CP-HMP-EDC and CPS-PPA-EDC in Figure 3).

Table 3 shows HMP to yield significantly larger ionotropic NPs compared to PPA and TPP, e.g., C-HMP NPs were of 459 nm average size, while C-PPA NPs and C-TPP NPs were of 118 and 205 nm average sizes, respectively. Similarly, CP-HMP NPs (415 nm) and CPS-HMP NPs (331 nm) significantly outsized their TPP and PPA counterparts: CP-TPP NPs (148 nm), CPS-TPP (156 nm), CP-PPA (133 nm), and CPS-PPA (120 nm). Moreover, HMP-based NPs remained larger than their TPP and PPA analogs after covalent crosslinking albeit at lesser size differences, e.g., CROSS-CPS-HMP NPs scored 254 nm average size at preparation pH, while CROSS-CP-TTP and CROSS-CP-PPA NPs scored 160 and 158 nm, respectively. We believe this trend is also related to our proposition that HMP is mainly core-ionotropic crosslinker, while TPP acts as surface (shell) crosslinker. Apparently, being surface crosslinker, TPP exerts electrostatic attraction against loose cationic chitosan layers directly beneath NPs surfaces leading to NPs size collapse, while core chitosan layers tend to be denser and harder to compress under the electrostatic influence of core HMP thus yielding larger NPs. PPA seems to act as both core/shell crosslinker, which also explains the smaller sizes of its corresponding NPs.

Table 3 shows lack of any trend connecting the pH with sizes or size distributions of covalent NPs. A similar conclusion can be drawn regarding the effect CaCl_2_ on covalent NPs sizes. However, PPA-based covalent NPs are a noticeable exception: CROSS-CP-PPA NPs increased in size from 158 to 471 nm upon exposure to CaCl_2_ (0.5 M)_._ We believe the long chains of covalently attached surface PPA allow polyphosphate strands to be involved in electrostatic attraction with surface chitosan ammonium groups (extra to those involved in covalent crosslinking). These strands are readily cleavable from their PPA mother chains under the acidic aqueous conditions experienced during NPs preparation leaving them electrostatically anchored to NPs surfaces. Higher calcium concentrations are expected to sequester these ionotropic polyphosphate strands leaving their covalently attached mother chains as sole crosslinking anchors thus relaxing the crosslinker strain at NPs surfaces and allow water diffusion into NPs interior leading to NPs size enlargement.

Table 4 shows NPs' zeta potentials and how they respond to ionotropic/covalent crosslinking and varying pH conditions. Clearly from the table, all ionotropically crosslinked NPs exhibit positive surface charges albeit significantly greater positive values are observed for NPs derived from unmodified chitosan. This is not surprising since grafting with anhydrides converts part of chitosan's surface cationic ammonium residues into neutral amides. Similar observations were reported previously (Yan et al., 2006; Dmour and Taha, 2017).

**Table 4:** Change of NPs zeta potentials upon covalent crosslinking and varying pH conditions.

**NPs ^a^^,^^b^**	**Zeta potential (mV)**					
	**Preparation pH^**d**^**	**pH 1.2^**d**^**	**pH 6.8^**d**^**	**pH 7.4**		**pH 12.0^**d**^**
				**Aqueous conditions**	**FBS (10% v/v)**	
C-TPP	+29.0 ± 1.31	Clear	Aggregate	Aggregate	—^e^	Aggregate
C-PPA	+25.5 ± 0.92	Clear	Aggregate	Aggregate	—^e^	Aggregate
C-HMP	+51.5 ± 1.08	+7.6 ± 0.73^c^	Aggregate	Aggregate	—^e^	Aggregate
CP-TPP	+14.7 ± 1.92	clear	Aggregate	Aggregate	Aggregate	Aggregate
CP-PPA	+11.0 ± 1.31	clear	Aggregate	Aggregate	Aggregate	Aggregate
CP-HMP	+36.8 ± 1.45	+19.0 ± 1.95^c^	Aggregate	Aggregate	Aggregate	Aggregate
CPS-TPP	+13.5 ± 0.72	Clear	Aggregate	Aggregate	Aggregate	Aggregate
CPS-PPA	+11.4 ± 1.05	Clear	Aggregate	Aggregate	Aggregate	Aggregate
CPS-HMP	+26.8 ± 1.57	Aggregate	Aggregate	Aggregate	Aggregate	Aggregate
CROSS-CP-TPP	+9.4 ± 0.41	+14.9 ± 2.90	+1.4 ± 0.16	−4.5 ± 0.47	−3.14 ± 0.58	−14.3 ± 0.50
CROSS-CP-PPA	+8.2 ± 1.19	+12.0 ± 0.80	−2.9 ± 0.80	−5.9 ± 0.95	−1.64 ± 0.36	−15.9 ± 0.80
CROSS-CPS-HMP	+20.0 ± 1.64	+16.0 ± 0.76	+2.2 ± 0.50	−5.1 ± 0.47	−7.07 ± 0.37	−12.0 ± 0.69

a *See Table 2 for pH value at preparation conditions*.

b *Each value represents the average of triplicate measurements ± standard deviation*.

c *NPs are stable without EDC*.

d *Aqueous conditions (no FBS)*.

e *Not tested*.

Interestingly though, HMP-based NPs (ionotropic and covalent) were accompanied with significantly higher positive surface charges (e.g., mean zeta potential for C-HMP NPs = +51 Mv) compared to their TPP- and PPA-based analogs (e.g., mean zeta potential of C-PPA NPs and C-TPP = +25.5 and 29 Mv, respectively).

This trend further proves our proposition that HMP is mainly core crosslinker with minimal influence on chitosan's cationic surface charge, while TPP, and partially PPA, act as surface crosslinkers that effectively neutralize positively charged chitosan's ammonium moieties by electrostatic attraction at NPs surfaces.

Table 4 points to another interesting observation by comparing zeta potentials of HMP-based ionotropic NPs, namely, CPS-HMP NPs and CP-HMP NPs. Clearly, CPS-HMP NPs exhibited significantly lesser surface charge (+26 Mv) compared to CP-HMP NPs (+36 Mv). Since both NPs formulations were crosslinked by the same core crosslinking agent (HMP) and they have virtually identical degrees of carboxylic acid substitution (as deduced from pH shifts upon ionotropic gelling, see Table 2), it can be firmly concluded that the difference in their surface charges is related to the relative distribution of carboxylic acid substituents (phthalic and phenylsuccinic acids) on NPs surfaces vs. cores. The lower positive surface charge of CPS-HMP NPs suggests higher concentration of phenylsuccinic acid substituents at NPs surfaces compared to phthalic acid residues in CP-HMP NPs, which seem to concentrate within NPs cores leaving NPs surfaces with more intense positive charge. Probably, this behavior is because phenylsuccinic acid substituents are more hydrophilic and prefer interaction with water molecules at NPs surfaces; while hydrophobic phthalic acid residues prefer NPs cores to minimize their interactions with aqueous surroundings.

This trend is not obvious in TPP and PPA-crosslinked NPs (i.e., CPS-TPP and CPS-PPA vs. CP-TPP and CP-PPA) because of the significant neutralization of surface charge affected by these shell crosslinkers (particularly TPP) leaving little opportunity for the subtle effects of carboxylic acid substituents on surface charge to be clearly evident.

Interestingly, Tables 3, 4 show covalent NPs to exhibit moderate size and surface charge changes upon exposure to FBS (i.e., compared to equivalent aqueous pH 7.4).

Success to achieve covalent crosslinking with certain ionotropic NPs (i.e., CP-TPP, CP-PPA, and CPS-HMP) and failure with others (i.e., CP-HMP, CPS-TPP, and CPS-PPA) prompted us to hypothesize that EDC coupling is dependent on the shell/core complementarity of phosphate crosslinker and grafted carboxylic acid: Covalent crosslinking succeeds only if the phosphate crosslinker and grafted carboxylic acid are of opposing core/shell distribution, while it fails if the polyphosphate/carboxylic acid combination exhibit similar core/shell distribution properties. For example, in the unsuccessful case of CP-HMP NPs both grafted phthalic acid residues and HMP reside mainly within NPs cores. It appears that the steric bulk of core phthalic acid residues hinder EDC-mediated coupling of chitosan amines with core HMP phosphate groups. Similarly, CPS-TPP and CPS-PPA failed the EDC crosslinking reaction because PPA, TPP, and the attached phenylsuccinic acid units concentrate at NPs surfaces causing the steric bulk of phenylsuccinic acid moieties to interfere with EDC-mediated phosphoramide formation reaction.

On the other hand, crosslinker/carboxylic, acid combinations of orthogonal core/shell distribution minimize any negative interference in EDC coupling reaction and thus lead to better chances of covalent crosslinking. For example, success in covalent crosslinking of CPS-HMP NPs (i.e., CROSS-CPS-HMP NPs) is because HMP molecules remain within NPs cores far from the steric influence of the surface phenylsuccinic acid residues leading to effective EDC-mediated coupling of core HMP with nearby chitosan amine moieties. In contrast, TPP and PPA in CP-TPP and CP-PPA NPs reside at NPs surfaces (shell crosslinkers) far from core phthalic acid residues allowing facile EDC-mediated phosphoramide coupling at NPs surfaces to give CROSS-CP-TPP and CROSS-CP-PPA.

Finally, Table 4 shows reduction in positive surface charges upon covalent crosslinking (from *ca*. +11 Mv for CP-PPA to +8 Mv for CROSS-CP-PPA; from *ca*. +14 Mv for CP-TPP to +9 Mv for CROSS-CP-TPP and from *ca*. +26 Mv for CPS-HMP NPs to +20 Mv for CROSS-CPS-HMP NPs) indicating that covalent crosslinking converted some surface chitosan ammonium moieties into neutral phosphoramides [and amides in CROSS-CPS-HMP NPs, see section Infrared spectroscopy (IR)**]** with loss of some positive surface charge.

#### NPs Morphology

**Figure 6:**
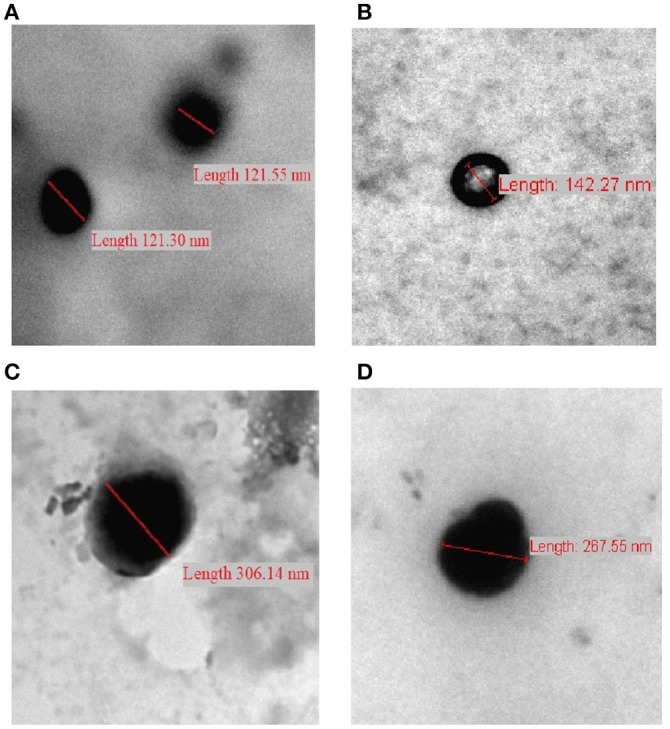
TEM images of **(A)** CP-PPA NPs, **(B)** CROSS-CP-PPA NPs, **(C)** CPS-HMP NPs, and **(D)** CROSS-CPS-HMP NPs.

We opted to use transmission electron microscopy (TEM) to assess the morphological properties of some representative NPs, as in Figure 6. The evaluated NPs have spherical shapes with sizes within the ranges identified by DLS. Covalent crosslinking did not change the morphology (i.e., spherical shapes) of the resulting NPs.

## Drug Loading and Release Profiles

Drug loading capacities (LCs) of ionotropic chitosan NPs depend on polyphosphate crosslinker content, chitosan-to-drug loading ratios (Wang et al., 2011a) and electrostatic interactions between the loaded drug(s) and the polymeric matrix of the NPs (Katas et al., 2013).

### Methylene Blue Loading Into NPs

Methylene blue (MB, Figure 1) was used as model drug to study the LCs of prepared NPs. Two polymer-to-MB loading ratios were studied, namely, 10:1 and 1:1. The LCs were determined by measuring the amounts of released MB following shaking MB-loaded NPs in TRIS buffer (pH 7.4) over 24 h at 100 rpm and 37°C. Core MB loadings that resisted release under these conditions were determined following acid-degradation of NPs. The resulting LCs are summarized in Table 5.

**Table 5:** LCs of MB-loaded NPs (mg/g).

**NPs**		**Total LC (mg MB per g polymer)**^****a****^			
**Crosslinker**	**Polymer**	**At 10:1 Polymer to MB ratio**		**At 1:1 Polymer to MB ratio**	
TPP	C	0.39 ± 0.05		7.33 ± 0.05	
	CP	1.97 ± 0.11	*p* = 0.0002^b^	18.31 ± 1.20	*p* = 0.0001^b^
	CROSS-CP^c^	4.57 ± 0.18		27.31 ± 0.62 (2.5 ± 0.05)^d^	
PPA	C	1.08 ± 0.02		10.87 ± 1.75	
	CP	2.02 ± 0.07	*p* = 0.0010^b^	37.19 ± 0.67	*p* = 0.030^b^
	CROSS-CP^c^	8.55 ± 0.35		62.05 ± 6.28 (3.9 ± 0.09)^d^	
HMP	C	1.33 ± 0.16		10.94 ± 0.96	
	CPS	4.63 ± 0.33	*p* = 0.9080^b^	100.30 ± 2.24	*p* = 0.022^b^
	CROSS-CPS^c^	4.66 ± 0.75		111.40 ± 1.58 (8.1 ± 0.02)^d^	

a *Each value represents the average of triplicate measurements± standard deviation*.

b *p-value Calculated using t-test with 5% significance for LC difference between covalent and corresponding ionotropic nanoparticles*.

c *EDC covalently stabilized NPs*.

d *NPs core loading determined through acid degradation (HCl, 2.0 M) of covalent NPs*.

Evidently from Table 5, MB loading increased significantly upon grafting chitosan with phthalic and phenylsuccinic acids. This trend is observed in ionotropic and covalent NPs alike. This behavior is not unexpected since the conjugated aromatic acids limit the cationic charge of chitosan, and therefore reduce electrostatic repulsion of cationic MB. Moreover, the aromatic rings of phthalic and phenylsuccinic conjugates provide viable flat surfaces for π-stacking interactions with MB thus promoting further NPs loading (Dmour and Taha, 2017). Additionally, grafted carboxylic acids act as hydrophobic barriers (being unionized under acidic conditions of NPs preparation) that limit free water exchange across NPs' surfaces thus hinder MB leaching from NPs during post loading processing (in particular centrifugation, see section Synthesis of chitosan-dicarboxylic acid derivatives and preparation of corresponding NPs).

Table 5 also shows another trend: Covalent crosslinking of CP NPs enhanced their LCs (i.e., in CP-TPP NPs from 18.3 to 27.3 mg/g, and in CP-PPA NPs from 37.2 to 62.1 mg/g). This is rather anticipated trend since covalent crosslinking makes NPs matrices stronger and more resistant to erosion, aqueous penetration and MB escape during processing steps performed after loading (Saboktakin et al., 2011; Dmour and Taha, 2017).

Nevertheless, ionotropic CPS-HMP NPs exhibited comparable LCs to their covalent counterparts CROSS-CPS-HMP NPs. Moreover, ionotropic and covalent CPS-HMP NPs illustrated the highest LCs amongst prepared NPs (at 1:1 loading ratios). We believe this behavior is because HMP attracts cationic MB molecules deeper into NPs cores during ionotropic NPs formation thus protecting loaded MB molecules from leaching into the medium during post loading processing. This mechanism seems to limit MB leaching from covalent CROSS-CPS-HMP NPs as well.

Interestingly, TPP-based NPs showed significantly lesser LCs compared to PPA and HMP-based counterparts. This is probably because PPA and HMP have more phosphate anions per molecule compared with TPP, which increase the efficiency of ionotropic binding with chitosan causing lesser leaching during NPs post loading processing.

### DOX NPs Loading, Release Profiles, and Cytotoxicities

DOX-loaded CP-PPA, CPS-HMP, CROSS-CP-PPA, and CROSS-CPS-HMP NPs were recruited to study NPs LCs, DOX release profiles, and cytotoxicities. These particular NPs formulas were selected to study DOX loading and release profiles because they achieved the best MB LCs (see Table 5). Table 6 shows their DOX LCs, average sizes and polydispersities.

**Table 6:** LCs and size properties of DOX-loaded NPs prepared by 1:1 polymer-to-DOX loading ratios.

**Ionotropic NPs**				**Covalent NPs**			
**NPs**	**Loaded Doxorubicin (mg/g NPs)^**a**^**	**Size (nm)^**a**^**	**PDI^a^^,^^b^**	**NPs**	**Loaded Doxorubicin (mg/g NPs)^**a**^**	**Size (nm)^**a**^**	**PDI^a^^,^^b^**
CP-PPA	149.20 ± 2.55	212.1 ± 4.71	0.33 ± 0.04	CROSS-CP-PPA	220.07 ± 1.38	314.8 ± 19.4	0.42 ± 0.16
CPS-HMP	143.70 ± 2.80	471.1 ± 15.9	0.48 ± 0.18	CROSS-CPS-HMP	174.67 ± 3.70	357.7 ± 12.3	0.44 ± 0.10

a *Each point represents at least duplicate measurements ± standard deviation*.

b *Polydispersity index*.

Obviously, comparing NPs sizes in Tables 3, 6 shows DOX-loaded NPs to have larger sizes compared to their unloaded counterparts. Unsurprisingly, Table 6 shows enhanced DOX LCs upon covalent crosslinking. Moreover, the tested NPs were able to load greater amounts of DOX compared to MB (Table 5) probably because MB is of higher water solubility [43.6 and 20 mg/ml for MB (Peters and Freeman, 1996) and DOX, respectively] leading to more MB loss during post loading processing.

**Figure 7:**
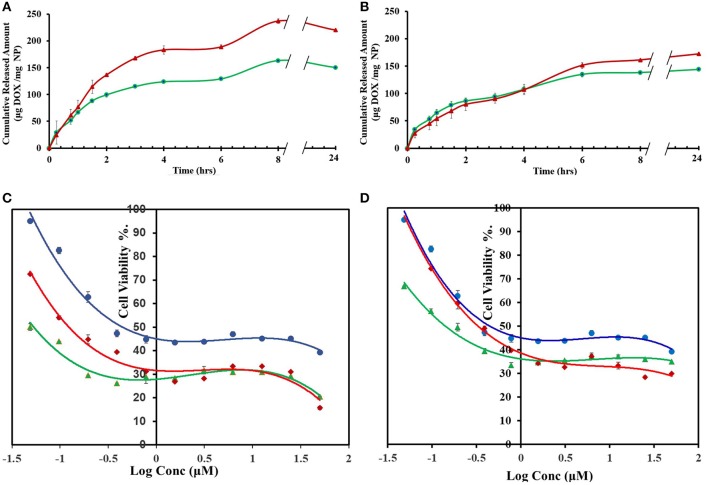
DOX release and cytotoxic properties of DOX-loaded NPs (formulated at 1:1 polymer to DOX ratios) **(A)** Cumulative amounts of DOX released from CP-PPA (green line, 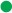) and CROSS-CP-PPA (red line, 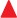). **(B)** Cumulative amounts of DOX released from CPS-HMP (green line, 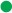) and CROSS-CPS-HMP (red line, 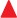). Dissolution studies were performed at 37°C and pH 7.4 TRIS buffer (100 rpm over 24 h) using 1:1 polymer to doxorubicin loading ratio. **(C)** MCF-7 cell viability after 72 h exposure to free DOX (blue line, 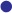), DOX-loaded ionotropic CP-PPA NPs (green line, 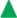) and DOX-loaded CROSS-CP-PPA NPs (red line, 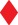). **(D)** MCF-7 cell viability after 72 h exposures to free DOX (blue line, 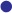), DOX-loaded ionotropic CPS-HMP NPs (green line, 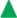) and DOX-loaded CROSS-CPS-HMP NPs (red line, 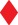). Each point represents the average of duplicate measurements. Error bars represent standard error of measurements.

DOX release profiles from CP-PPA, CPS-HMP, CROSS-CP-PPA, and CROSS-CPS-HMP NPs are shown in Figures 7A,B. Three release phases can be recognized in the figure (seen in all tested NPs formulations): An initial fast phase (burst release) during the first 45 min resulting from quick dissolution of DOX molecules loosely adsorbed at NPs surfaces. A second slower subsequent phase, extending over 2–3 h, probably associated with water penetration through NPs matrices. A third phase, after ~4–6 h, believed to be due to the degradation of polyphosphate crosslinkers (PPA and HMP) releasing DOX molecules deeply entrenched within NPs matrices.

The anticancer properties of DOX-loaded CP-PPA, CPS-HMP, CROSS-CP-PPA, and CROSS-CPS-HMP NPs were evaluated against breast cancer MCF-7 cells, which are widely used for assessing DOX drug delivery systems (Naruphontjirakul and Viravaidya-Pasuwat, 2019; Zhong et al., 2019). Unloaded NPs were virtually non-cytotoxic with cell viabilities exceeding 97% after exposure over 72 h. On the other hand, unloaded C-PPA and C-HMP NPs show significant cytotoxic properties with cell viabilities of *ca*. 80% upon exposure over the same time interval. Chitosan cytotoxicity is related to its cationic nature which disrupts the architecture of intercellular junctions between cancer cells (Loh et al., 2010; Fröhlich, 2012; Chokradjaroen et al., 2018; Morovati et al., 2019). Grafting chitosan with phthalic or phenylsuccinic acid reduce the cationic nature of chitosan and thus minimize the cytotoxicities of corresponding NPs.

Table 7 and Figures 7C,D show the anticancer profiles of DOX-loaded NPs compared to free DOX. The anticancer IC_50_ of DOX was enhanced by factors of 10 and 3.3 times upon loading in CP-PPA and CROSS-CP-PPA NPs, respectively (Table 7). This result suggests that loaded NPs are more efficiently up-taken by cancer cells compared to free DOX thus leading to higher intracellular DOX concentrations and cell death at lesser IC_50_ values. We believe this cytotoxic enhancement is due to the favorable sizes of loaded CP-PPA and CROSS-CP-PPA NPs (Table 6). Nevertheless, ionotropic CP-PPA performed better than their covalent counterparts (CROSS-CP-PPA) probably because they dissolve upon entry into cancer cells releasing all their DOX content, while their covalent siblings resist complete breakdown within cancer cells causing lesser intracellular release of DOX.

**Table 7:** IC_50_ of free DOX and DOX-loaded NPs against MCF-7 cell line.

**Treatment**	**${\text{IC}}_{\text{50}}^{\text{a}}$(μM)**
Free DOX	0.457 ± 0.039
DOX loaded CP-PPA NPs	0.048 ± 0.001
DOX loaded CROSS-CP-PPA NPs	0.139 ± 0.014
DOX loaded CPS-HMP NPs	0.124 ± 0.005
DOX loaded CROSS-CPS-HMP NPs	0.360 ± 0.028

^a^*Each value represents the average of duplicate measurements ± standard deviation. Figure S30 shows the cytotoxic effects of DOX and DOX-loaded NPs on tested cells*.

Regarding CPS-HMP NPs and their covalent progenies (CROSS-CPS-HMP NPs), they seem to have inferior anticancer performances compared to their ionotropic and covalent CP-PPA NPs analogs. We believe this difference in performance is related to the lesser LCs and enhanced physical and chemical stabilities of CPS-HMP NPs (ionotropic and covalent) compared to their CP-PPA analogs, which seem to reduce the rate of DOX release inside cancer cells.

To investigate NPs uptake by MCF-7 cells we used confocal laser scanning microscopy and wide-field fluorescence microscopy as means to compare cellular uptake of DOX-loaded NPs compared to free DOX. Untreated cells were evaluated as controls.

**Figure 8:**
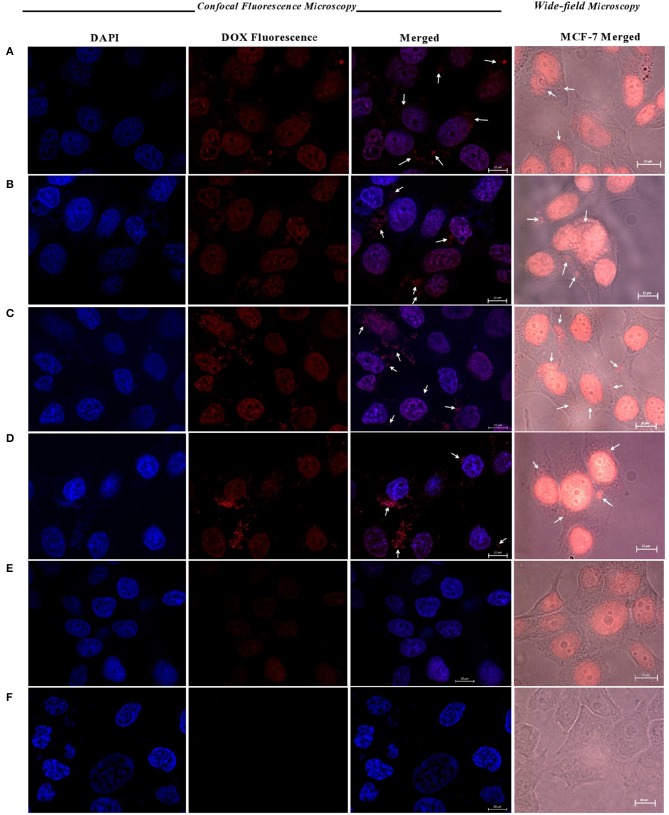
Confocal and wide-field fluorescence microscopy images showing MCF-7 cells treated with DOX-loaded **(A)** CP-PPA NPs, **(B)** CROSS-CP-PPA NPs, **(C)** CPS-HMP NPs, **(D)** CROSS-CPS-HMP NPs, **(E)** MCF-7 cells treated with free DOX, and **(F)** untreated cells (control). All treatments are equivalent to 1.0 μM doxorubicin over 4 h periods. White arrows point nanoparticles up taken into cellular cytoplasm. Scale: 10 μm.

Figure 8 shows wide-field fluorescence microscopy images of MCF-7 cells treated with CROSS-CP-PPA and CROSS-CPS-HMP NPs compared to free DOX. The images clearly demonstrate the internalization of DOX-loaded NPs within cellular cytoplasm. Figure 8 further illustrates the cellular uptake of loaded NPs with crystal-clear resolution using confocal fluorescence microscopy, particularly upon staining with DAPI to distinguish cellular nuclei from cytoplasms. In contrast to untreated cells, DOX-loaded NPs and free DOX caused cellular nuclei to fluoresce indicating nuclear uptake of DOX. However, cells treated with DOX-loaded NPs exhibited significantly more intense fluorescence compared to free DOX-treated cells indicating more efficient DOX cellular uptake via NPs. This is not unexpected since MCF-7 cells are known to exhibit DOX resistance via P-glycoprotein efflux pump (Wu et al., 2011). On the other hand, DOX-loaded NPs are not appropriate substrates for the efflux pump process (due to excessive large size), allowing efficient entry of NPs with their DOX cargos. This conclusion is supported by the appearance of numerous fluorescent aggregates within cellular cytoplasm after exposure to DOX-loaded NPs, as in Figure 8. These observations support our cytotoxicity results in Table 7 and Figures 7C,D, suggesting that our NPs allow efficient entry of DOX into MCF-7 cells leading to improvement in DOX IC_50_ values (Table 7).

## Conclusions

Lack of sufficient stability of chitosan NPs prompted us to produce novel stable chitosan NPs suitable for drug delivery applications. Chitosan was first grafted to phthalic or phenylsuccinic acids. Subsequently, PPA, HMP, or TPP were used to achieve tandem ionotropic/covalently crosslinked chitosan NPs in the presence of EDC. Infrared and DSC analysis confirmed the formation of phosphoramide bonds between chitosan and polyphosphate crosslinkers within NPs matrices. DLS and TEM size analysis indicated spherical NPs with size range below 350 nm. The generated NPs exhibited excellent stabilities under variable pH and CaCl_2_ concentrations.

DLS, NPs stability and IR data suggest HMP to reside within NPs cores, while TPP and PPA act mainly as surface crosslinkers. However, we cannot exclude the possibility of certain degree of surface crosslinking by HMP and/or bulk crosslinking in TPP and PPA cases.

Drug loading and release studies using methylene blue (MB) and doxorubicin (DOX) drug models showed covalent PPA- and HMP-based NPs to have superior loading capacities compared to NPs based on unmodified chitosan, generated by ionotropic crosslinking only or covalently crosslinked by TPP. DOX-loaded CP-PPA NPs exhibited 10-fold cytotoxicity enhancement compared to free DOX.

Despite their success in delivering DOX into cancer cells, our new chitosan-based NPs need to be fully investigated with regard to biodegradability and elimination to be successfully implemented within clinical settings. We are currently researching these issues.

## Data Availability Statement

The datasets generated for this study are available on request to the corresponding author.

## Author Contributions

MT: study conception and administration. MT, RS, and ID: methodology and validation. RS: experimental work and manuscript drafting. MT and RS: manuscript review and editing.

### Conflict of Interest

The authors declare that the research was conducted in the absence of any commercial or financial relationships that could be construed as a potential conflict of interest.
