# Supplementary material for: Stable Chitosan-Based Nanoparticles Using Polyphosphoric Acid or Hexametaphosphate for Tandem Ionotropic/Covalent Crosslinking and Subsequent Investigation as Novel Vehicles for Drug Delivery
Source: Front Bioeng Biotechnol. 2020 Jan 24;8:4. doi: 10.3389/fbioe.2020.00004 (PMC6993129; doi:10.3389/fbioe.2020.00004)
Supplement: Supplementary file 1 [file Table_1.DOC]

Supplementary Materials

Stable Chitosan-Based Nanoparticles Using Polyphosphoric Acid or Hexametaphosphate for Tandem Ionotropic/Covalent Crosslinking and Subsequent Investigation as Novel Vehicles for Drug Delivery.

RamziMukred Saeed^1^, Isra Dmour^2^, Mutasem O Taha^1*^

^1^Department of Pharmaceutical Sciences, Faculty of Pharmacy, University of Jordan, Amman, 11942 Jordan

^2^Faculty of Pharmacy and Medical Sciences, Al-AhliyyaAmmanUniversity, Amman, Jordan

*Corresponding author: email address: mutasem@ju.edu.jo

**Figure S1:** Proposed mechanism for the heat-induced phosphoramide formation reaction between chitosan's amine groups and polyphosphoric acid moieties.


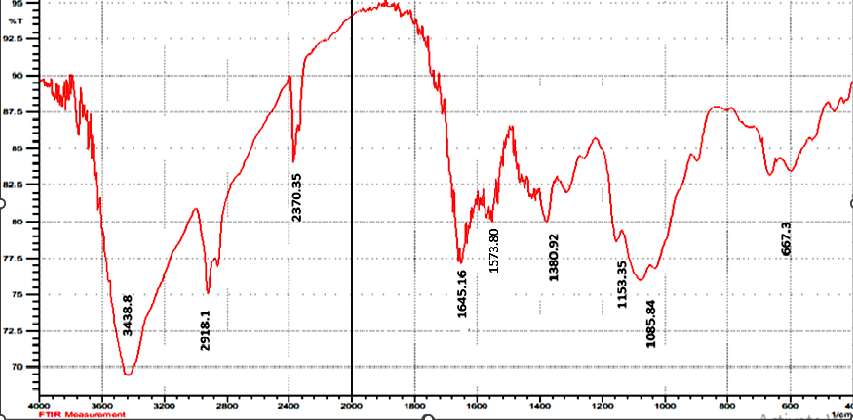


**Figure S2:** Infrared of Chitosan (C).


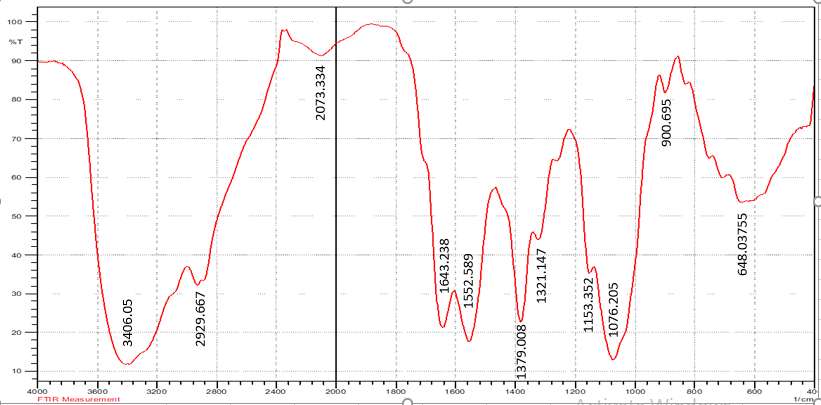


**Figure S3:** Infrared of Chitosan Phthalate (CP(.

**Figure S4:** Infrared of Chitosan Phenylsuccinate (CPS).


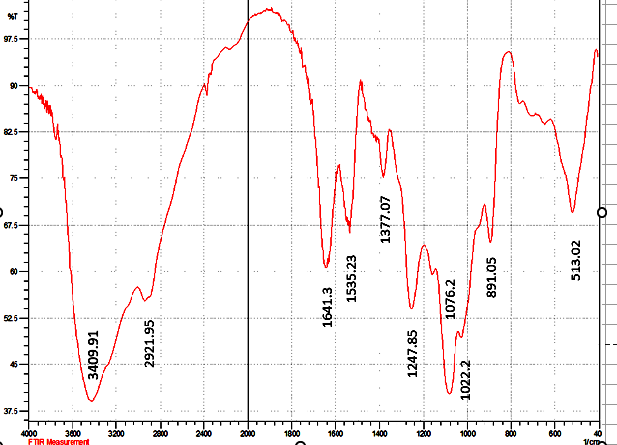


**Figure S5:** Infrared of C-PPA NPs.

**Figure S6:** Infrared of Chitosan polyphosphoric acid ionotropically crosslinked NPs after the addition of EDC (C-PPA-EDC).


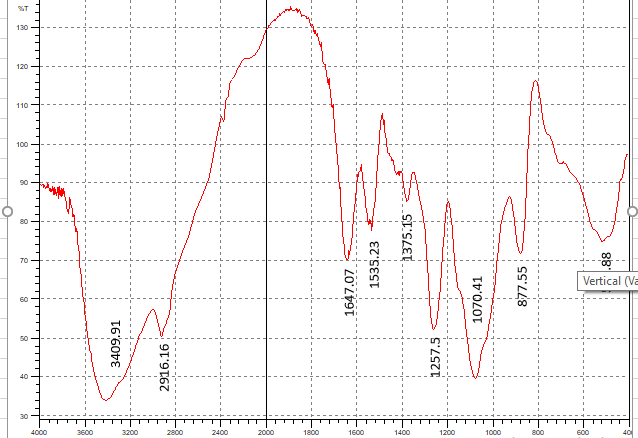


**Figure S7:** Infrared of C-HMP NPs.

**Figure** **S8:** Infrared of Chitosan Hexametaphosphate Ionotropically Crosslinked NPs after the addition of EDC (C-HMP-EDC).


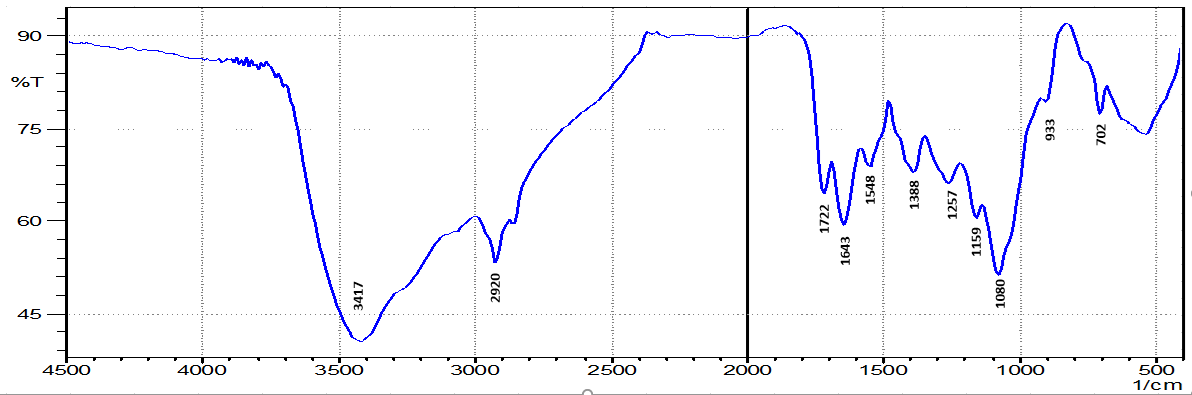


**Figure S9:**Infrared of CP-HMP NPs.


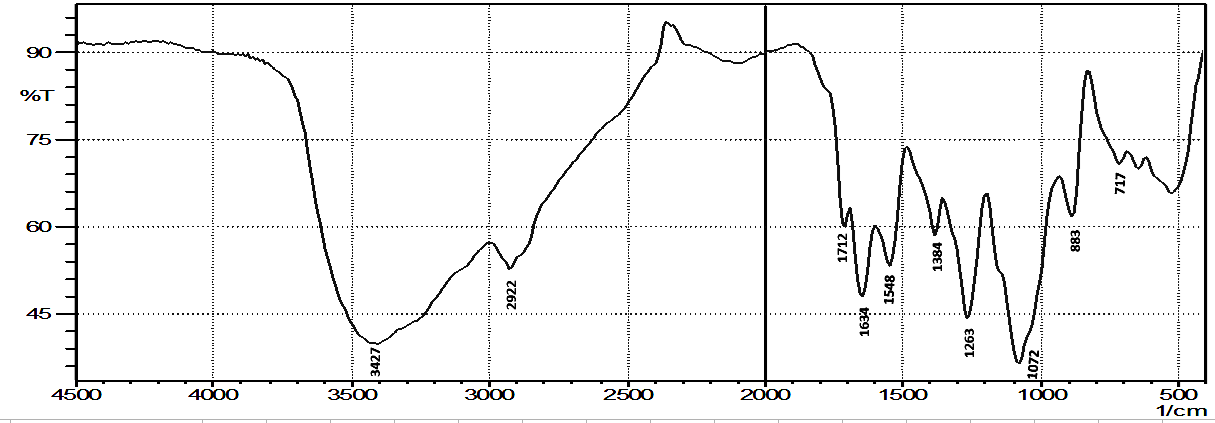


**Figure S10:** Infrared of Chitosan Phthalate Hexametaphosphate Ionotropically crosslinked NPs after addition of EDC (CP-HMP-EDC).

**Figure S11:** Infrared of CPS-PPA NPs.

1241.2

1540.1

3320.21

1080.2

1709.2

**Figure S12:** Infrared of Chitosan Phenylsuccinate polyphosphosric acid ionotropic NPs after EDC addition (CPS-PPA-EDC).

1080.3

1640.9

1710.2

3280.29

1241.6

**Figure S13:** Infrared of CP-PPA NPs.

**Figure S14:** Infrared of CROSS-CP-PPA NPs.

**Figure S15:** Infrared of CPS-HMP NPs.

**Figure S16:** Infrared of CROSS-CPS-HMP NPs.


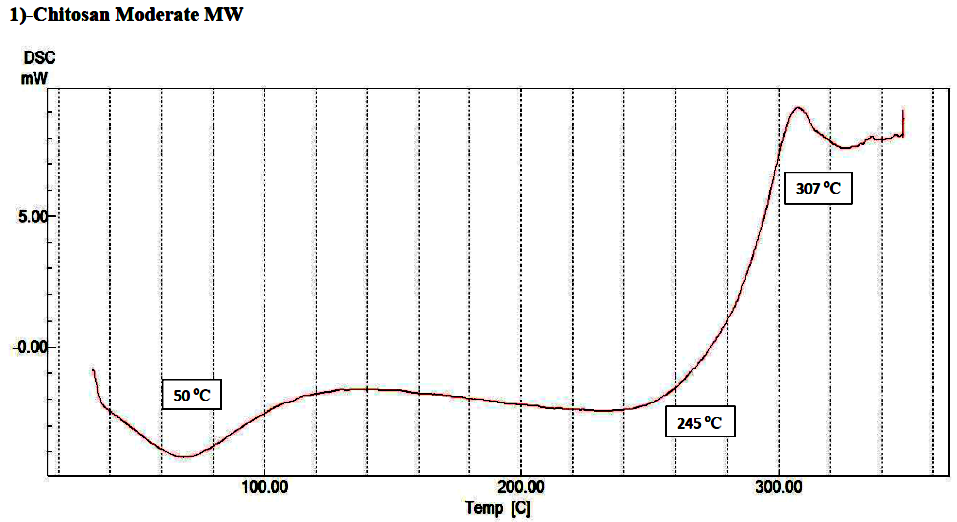


**Figure S17:** DSC of Chitosan (C).


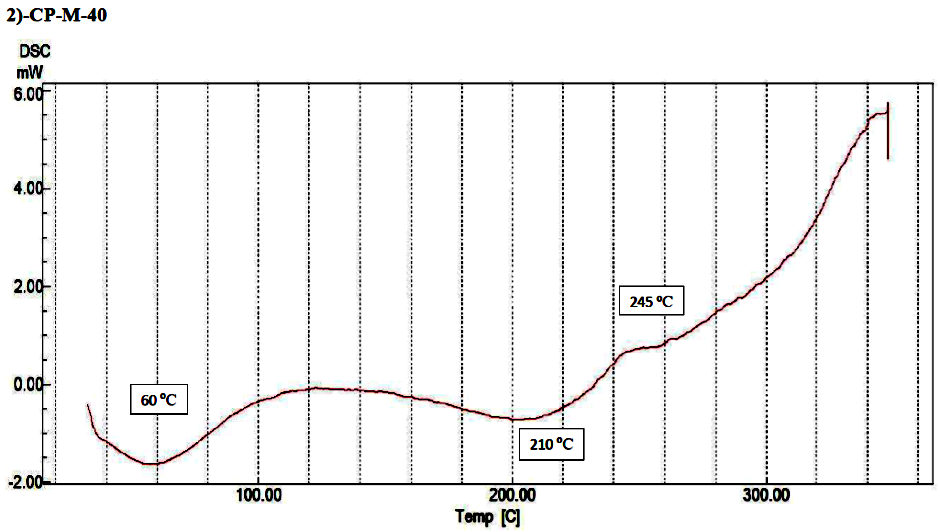


**Figure S18:** DSC of Chitosan Phthalate (CP(

**
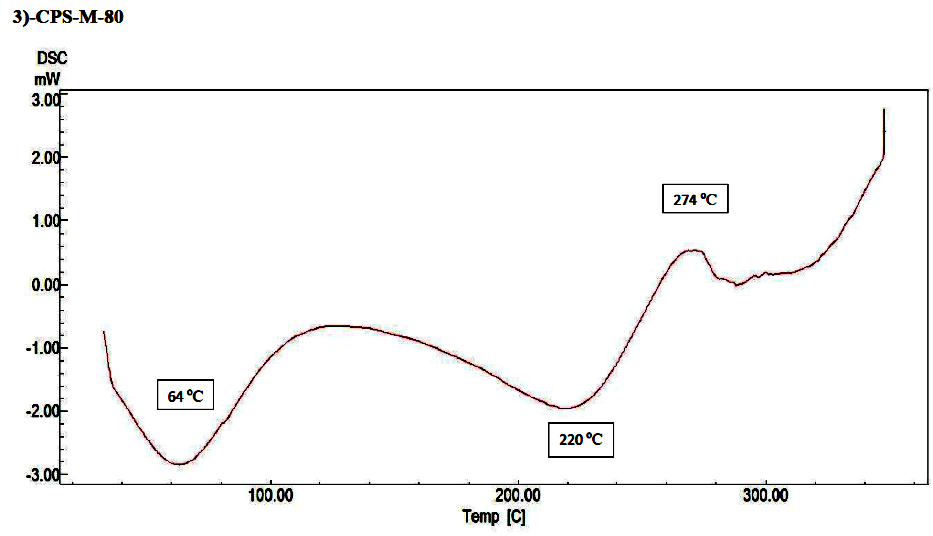
**

**Figure S19:** DSC of Chitosan phenylsuccinate (CPS).

**
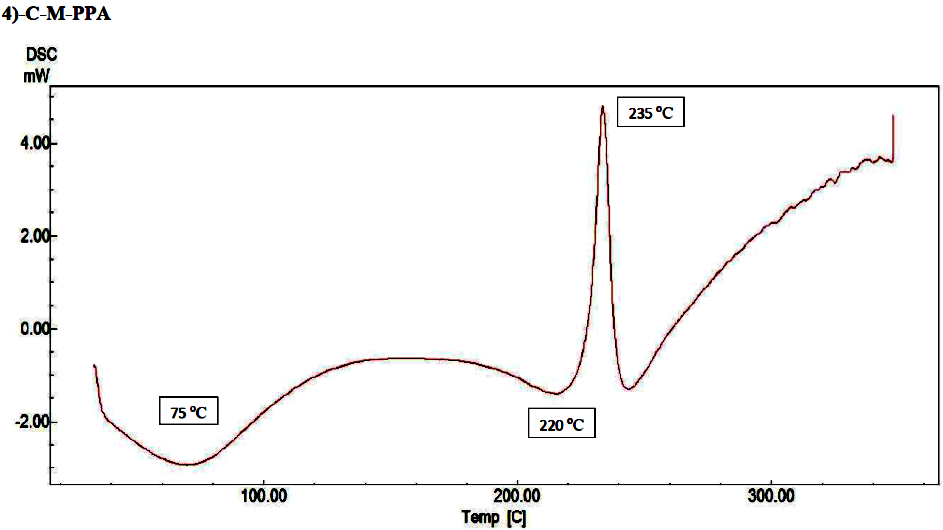
**

**Figure S20:** DSC of C-PPA NPs.

**
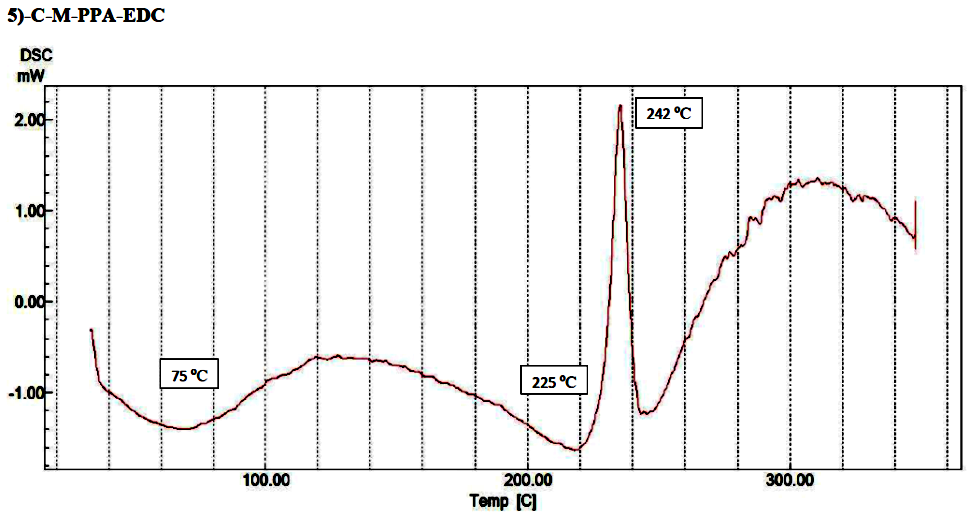
**

**Figure S21:** DSC of Chitosan Polyphosphoric Acid Ionotropically Crosslinked NPs after the addition of EDC (C-PPA-EDC).

**
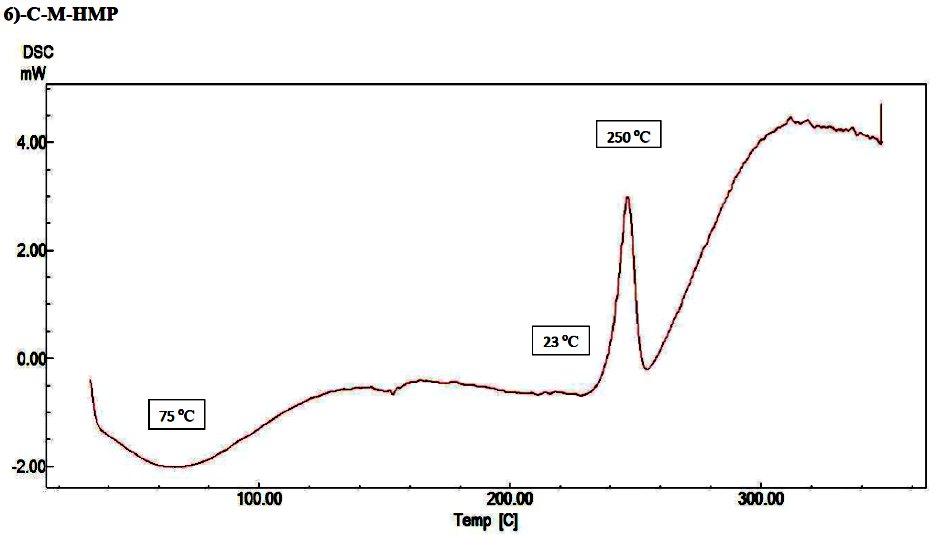
**

**Figure S22:** DSC of C-HMP NPs.

**
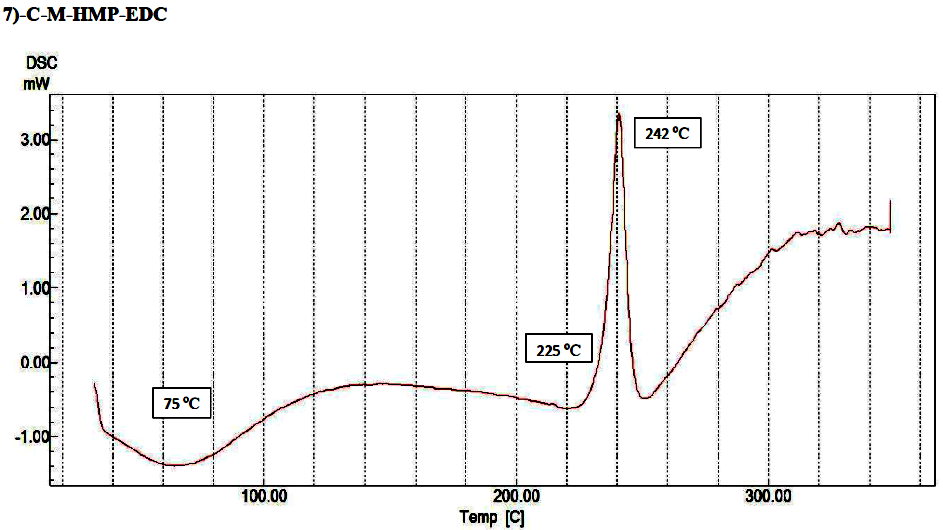
**

**Figure S23:** DSC of chitosan-hexametaphosphate ionotropically crosslinked NPs after the addition of EDC (C-HMP-EDC).

**
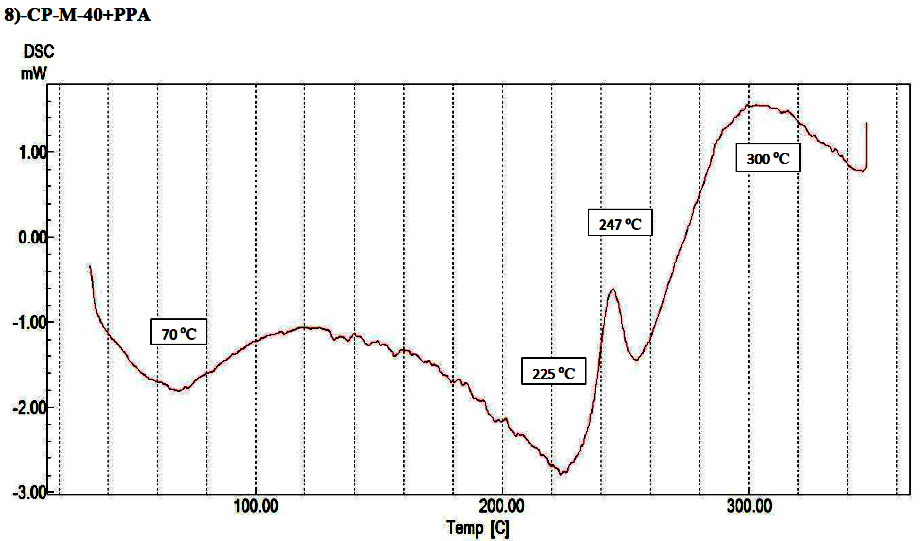
**

**Figure S24:** DSC of CP-PPA NPs.

**
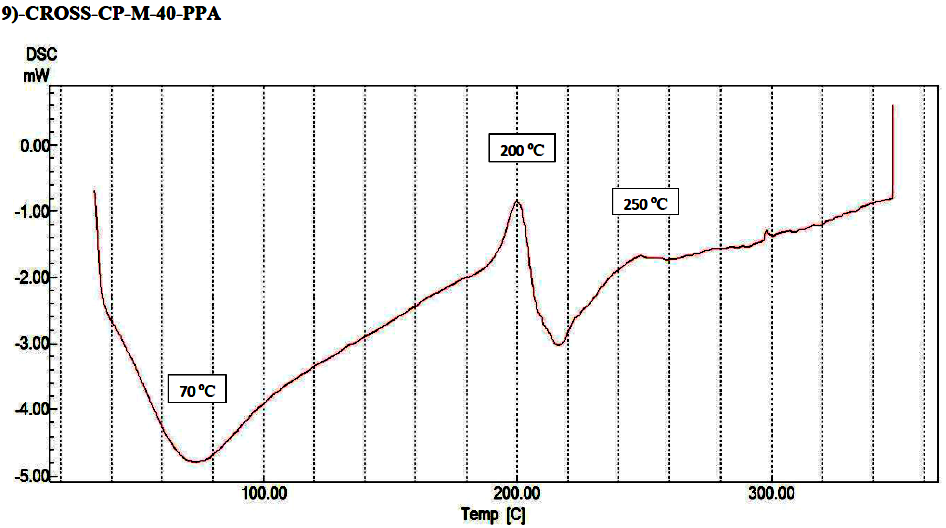
**

**Figure S25:** DSC of CROSS-CP-PPA NPs.

**
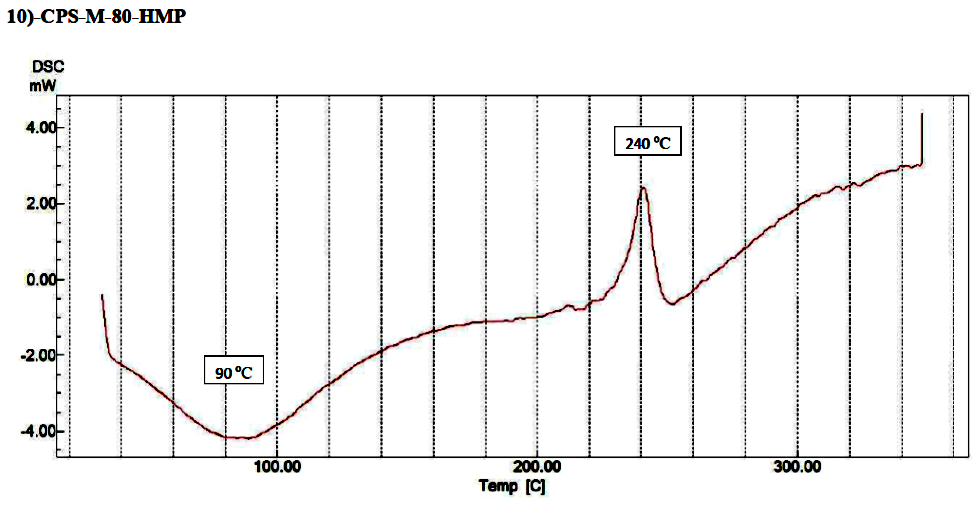
**

**Figure S26:** DSC of CPS-HMP NPs.

**
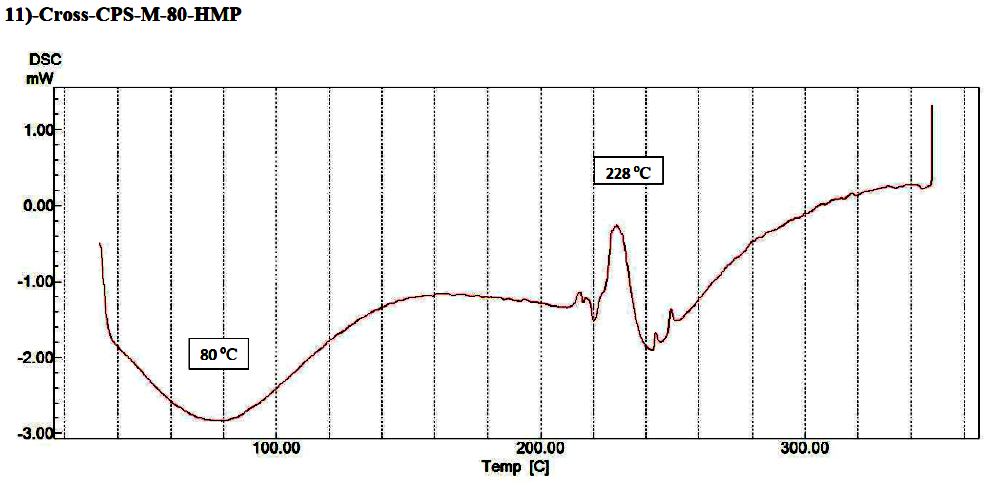
**

**Figure S27:** DSC of CP-HMP NPs.

**(A)**

**(B)**

**(C)**

**Figure S28:** Example dynamic light scattering (DLS) size distribution Graphs

of: (A) CPS-TPP, (B) CP-PPA, and (C) CPS-HMP. Each graph represents triplicate measurements (each measurement is represented with a different line)**.**

**Figure S-29** ^1^H NMR spectrums of **(A)** chitosan, **(B)** chitosan phenylsuccinate, and **(C)** chitosan phthalate.

Signals of polymeric protons

**(A)**

Signals of CH_2_ protons

**(B)**

Signals of aromatic protons

Signals of polymeric chitosan protons

**(C)**

Signals of polymeric chitosan protons

Signals of aromatic protons


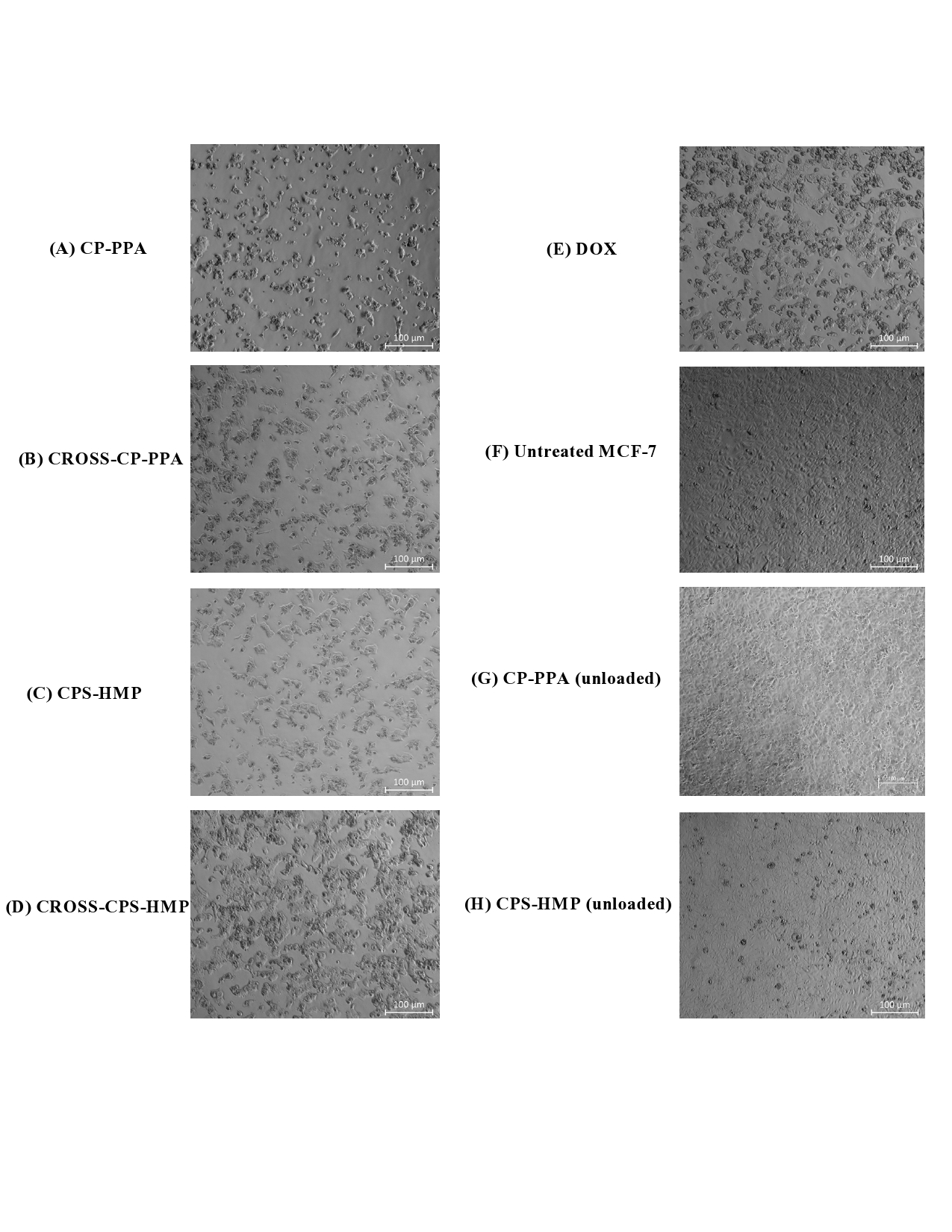


**Figure S-30:** Inverted light microscopy images of MCF-7 cytotoxicity after 72 h incubation with **(A-D)** DOX-loaded NPs, **(E)** free DOX, **(F)** untreated cells, **(G)** CP-PPA NPs free of DOX and **(H)** CPS-HMP NPs free of DOX. All treatments are equivalent to 10 μM doxorubicin. Scale: 100 μm.
